# Livestock Farm Recovery Following Bushfire in South-Eastern Australia: Impacts on Cattle and Sheep Health and Management

**DOI:** 10.3390/ani15121764

**Published:** 2025-06-14

**Authors:** Megan Thomas, John Webb Ware, Brendan Cowled, Carolina Munoz, Elicia Cheah, Peter Mansell, Henry Clutterbuck, Mark Doyle, Alison Hillman, Caitlin Pfeiffer

**Affiliations:** 1 Melbourne Veterinary School, Faculty of Science, University of Melbourne, Parkville 3010, Australia; megan.thomas@unimelb.edu.au (M.T.); j.webbware@unimelb.edu.au (J.W.W.); munoz.c@unimelb.edu.au (C.M.); pmansell@unimelb.edu.au (P.M.); 2 Ausvet, Fremantle 6160, Australia; brendan@ausvet.com.au (B.C.); alison.hillman@ausvet.com.au (A.H.); 3 Sydney School of Veterinary Science, Faculty of Science, University of Sydney, Camden 2050, Australia; 4 South East Local Land Services, Goulburn 2580, Australia

**Keywords:** bushfire, livestock, cattle, sheep, Australia, animal health, farm management, burns, biosecurity

## Abstract

Severe bushfires in South-Eastern Australia during the summer of 2019/2020 killed tens of thousands of livestock, while many more survived on fire-affected properties. At the time, the literature on bushfire and livestock in Australia primarily described animals with burns. The mid- to long-term health effects, if any, of fire exposure and the effectiveness of recovery activities for surviving animals and farm enterprises were rarely reported. This study aimed to describe the key impacts of bushfire exposure on the health, welfare, and management of surviving cattle and sheep to inform recommendations for future fire-affected farmers and to guide future research. As part of a broad research programme, data on bushfire experiences, consequences for the livestock and the farm enterprise, and lessons learnt were gathered through face-to-face interviews and an online survey, collating information from 58 fire-affected farmers. A variety of health conditions at low frequency were reported in livestock surviving bushfires, including respiratory disease, eye disease, ruminal acidosis, lameness, and plant toxicities. No single disease was observed widely across participating farms, with many conditions likely associated with management changes post-fire rather than direct fire exposure. A variety of management and farm biosecurity challenges were also described by farmers. Key strategies for the prevention and treatment of health conditions in livestock post-bushfire include supporting effective farm management through the challenges of post-fire recovery and early detection for treatment on a case-by-case basis.

## Simple Summary

Severe bushfires in South-Eastern Australia during the summer of 2019/2020 (“Black Summer”) killed tens of thousands of livestock, and many more survived on fire-affected farms. At that time, there was little known about the impact of fire on the surviving livestock or farm recovery in the aftermath of a bushfire in Australia. This study aimed to describe the impacts of the 2019–2020 bushfires on the health, welfare, and management of the cattle and sheep to inform recommendations for future fire-affected farmers and guide further research. Fifty-eight fire-affected farmers participated in either a face-to-face interview or an online survey to collect information about their bushfire experiences, observations of livestock health and welfare following the fire, and farm recovery activities. A range of health conditions were reported at low levels in surviving livestock, including respiratory disease, eye disease, ruminal acidosis, lameness, and plant toxicities. No single disease was observed widely across participating farms, with many conditions likely associated with management changes post-fire rather than direct fire exposure. A variety of management and farm biosecurity challenges were described by farmers. Strategies for the prevention and treatment of health conditions in livestock following a bushfire include supporting effective farm management through the challenges of recovery and early detection for treatment as needed.

## 1. Introduction

During the summer of 2019/2020 in southern Australia, the largest recorded bushfire event in Australia’s modern history occurred, now known as “Black Summer” [1]. These bushfires had devastating effects on many farms, with estimates of more than 56,000 livestock killed or euthanised in New South Wales (NSW), Victoria, and South Australia [2], in addition to other widespread impacts on the human population [3]. Despite the consistent presence of bushfires in the Australian landscape, there is very limited structured research describing the effects of bushfire on livestock health and productivity, though some systematic assessments have recently occurred examining the pathology of burns in livestock and the management of bushfire-injured livestock [4]. Despite this, a clearer understanding of a broader range of effects and the severity of these effects could support animal health professionals and livestock industry bodies to provide evidence-based advice to farmers, so they can mitigate any ongoing impacts of bushfire beyond the immediate post-fire period.

The existing literature on bushfire (or wildfire) and livestock in Australia primarily describes livestock with burns, predominantly through case studies [5,6,7,8,9,10,11,12,13,14,15,16,17,18]. Pneumonia, unexplained deaths, and reproductive losses have been reported in cattle, sheep, and goats following a fire season in the United States of America [19]. In addition to the direct effects of fire and smoke, other aspects of bushfires may impact the health, welfare, and management of livestock, with examples listed in Table 1. Some effects may be counterintuitive, for example, infection pressure for gastrointestinal nematodes may be lower due to a reduced environmental nematode load in the aftermath of fire [20]. The impacts of these factors have not been reported in detail, and the mid- to long-term health effects, if any, of fire exposure are poorly documented.

In Australia, a range of documents containing information that may aid farmers in the recovery period following a bushfire are available from government animal health agencies and livestock industry bodies. These provide advice regarding the feeding and care of surviving animals [25,26,27,31,34,35,36,37,38,39], the disposal of dead stock [40,41,42], and to a more limited extent, the recovery of pasture [43,44]. However, there is little evidence for whether the available advice is practical in the management of the health, welfare, and productivity of livestock on fire-affected properties, how effectively this advice is implemented, and what farmers actually do in the aftermath of a fire.

The climatic trend towards a greater number of high-fire-risk days and lengthened fire seasons in southern Australia [45] and globally [46] suggests an increasing need for evidence-based advice for livestock farmers recovering from bushfires. Therefore, as part of a broader project, detailed information on livestock health, welfare, and farm recovery activities was sought from livestock farmers whose land was burnt during the Black Summer. The objectives of this study were as follows:

1. Document the farmers’ observations of animal health and the welfare impacts of bushfire on livestock, including deaths due to fire injury, the subsequent observations of disease, negative reproductive outcomes and poor health post-fire, and the effects of loss of farm infrastructure;
2. Describe the implementation of biosecurity measures by fire-affected farmers and the impacts of bushfire on livestock farm biosecurity;
3. Describe the recovery activities of and ‘lessons learned’ by interviewed farmers.

The aim was to describe the key negative impacts of bushfire on animal health, welfare, and biosecurity, based on farmer interviews and surveys, to inform recommendations to future fire-affected farmers and guide future research. In addition, the ‘lessons learned’ by the interviewed farmers have contributed directly to the development of a ‘bushfire preparation and recovery manual’ for livestock farmers [47].

## 2. Materials and Methods

Data were collected through interviews with fire-affected farmers and an online survey of fire-affected farmers, each targeting similar farmer populations.

### 2.1. Interviews with Fire-Affected Farmers

#### 2.1.1. Study Area and Farm Recruitment

**Figure 1:**
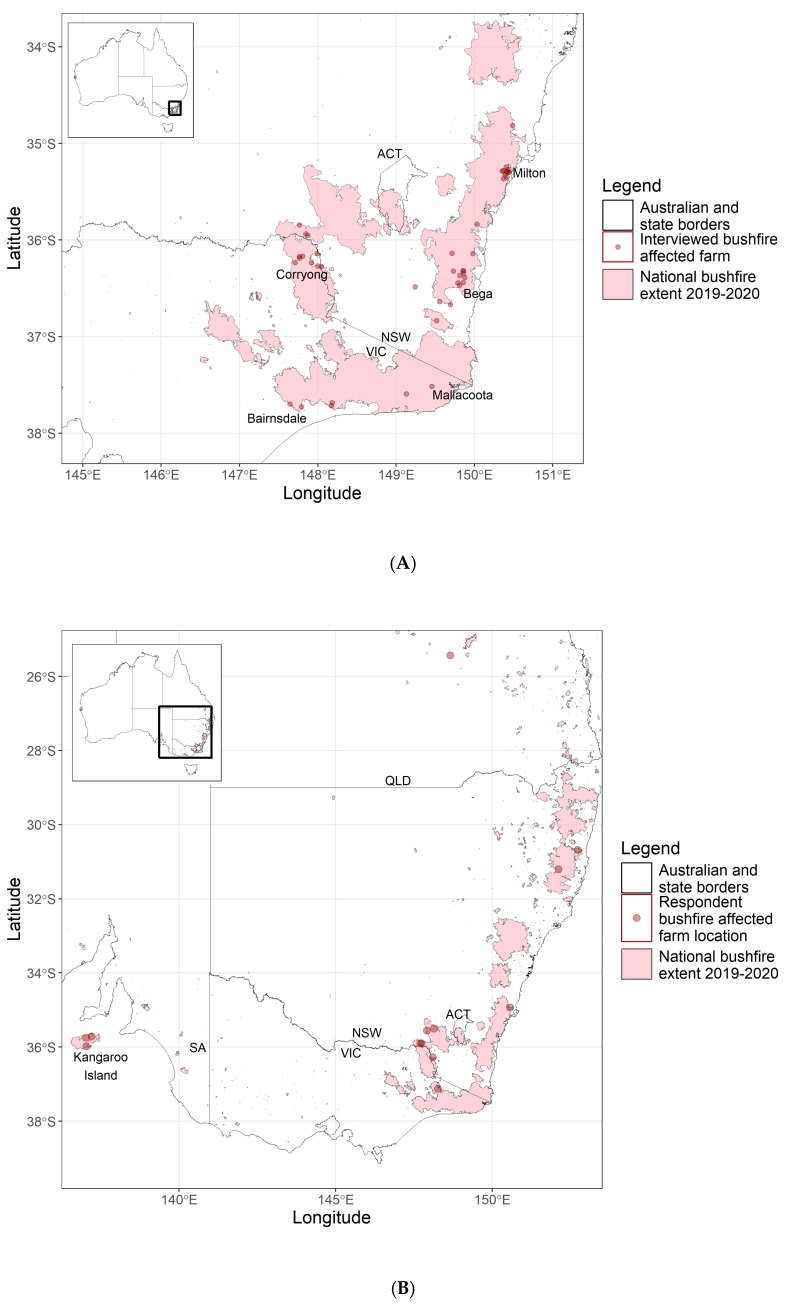
Locations of fire-affected farms (red points) in Australia participating in interviews (**A**) or an online survey (**B**) to describe farmers’ observations of livestock health and welfare, biosecurity, farm impacts, and recovery in the months following bushfires in 2019–2020. Pink shaded areas indicate the extent of land burnt by bushfires in 2019–2020. The arrow indicates the north direction. ACT = Australian Capital Territory, NSW = New South Wales, QLD = Queensland, SA = South Australia, and VIC = Victoria. Figure adapted from Cowled et al., 2022 [48].

The data presented were collected during interviews to inform a broad project, including both a case–control study to compare outcomes between farms with and without burnt livestock [48] and this descriptive study. The project studied a bushfire-affected region with a total land area of 9.1 million ha of which 3 million ha was burnt, spanning South-East NSW (comprising the NSW South-East region and the Eastern part of the Murray region, as defined by NSW Local Land Services) and Northern and Eastern Victoria (comprising the local government areas of East Gippsland shire and Towong shire). This region (Figure 1A) was selected purposively as it was the main bushfire-affected area in South-Eastern Australia between November 2019 and January 2020.

Farms with cattle (either dairy or beef) that had bushfire anywhere on the farm between November 2019 and January 2020 were eligible for inclusion in this study. Some of these farms also had sheep. In NSW, recruitment was supported by the Local Land Services (LLS) district veterinarians responsible for each target district (Bega, Milton, and Cooma). These veterinarians were consulted in August 2020, approximately 7 months after the fires ceased, and attempted to identify all fire-affected farms in each district, based on veterinarian recall and diary entries. Fire-affected farms known to have burnt livestock were invited to participate in this study by the LLS veterinarians. An additional equal number of fire-affected farms without bushfire-injured livestock were randomly selected from the same district and were invited to participate. In effect, all fire-affected landholdings in the region that contained livestock (beef and dairy cattle, including some mixed sheep and beef farms) were therefore eligible, and the sampling frame was the list of farms held by NSW LLS in the southeast of NSW [48].

A similar approach was taken in East Gippsland (Victoria) and the Upper Murray area (Victoria–NSW border), except private veterinarians rather than district vets were consulted to identify their clients with burnt farms to provide farms with and without burnt livestock in October and November 2020 (9–10 months after fires finished). The private veterinarians initially contacted eligible client farms non-randomly based on the vet’s perceptions of farmer willingness to participate. The details of consenting farms were subsequently provided to the research team. In Upper Murray, fire-affected farms with burnt livestock and an additional equal random sample of farms without bushfire-injured livestock in the district were invited to participate. In East Gippsland, only farms without bushfire-injured livestock could be successfully recruited [48].

#### 2.1.2. Interview Questionnaire Design and Data Collection

Data were collected through an interview on each participating fire-affected farm using a structured survey. In most cases, interviews occurred face to face and were delivered by a veterinarian attending the farm who also collected samples from fire-exposed cattle. In a small number of cases, interviews occurred by phone or video conferencing due to COVID-19 restrictions or farmer preference. Computer-aided delivery and recording of responses were used for all interviews using Qualtrics software (https://www.qualtrics.com, version July 2020), resulting in a digital database of all interview data. Interviews were conducted between July 2020 and January 2021. The questionnaire is available in full in the Supplementary Materials (S1).

The questionnaire focused on several areas:

- Pre-interview eligibility checks;
- Bushfire presence, cause, and temporal aspects;
- Fire intensity;
- Fire history, topography, and weather conditions;
- Farm type and management;
- Preparation for fire;
- Fire response;
- Farm impacts;
- Fire recovery;
- Burnt livestock and other health impacts;
- Livestock reproduction and management calendar;
- Livestock nutrition;
- Biosecurity and disease;
- Respondent demographic and contact details.

This descriptive study focussed on analysing a subset of the questionnaire data: animal health and welfare, biosecurity, fire recovery, and farm impacts. The analysis of risk factors for livestock bushfire injuries (burns) or death is presented separately [48]. There were no missing or incomplete data in the responses collected in the farmer interviews.

### 2.2. Online Survey of Fire-Affected Farmers

#### 2.2.1. Survey Design

This survey was designed as a cross-sectional study to supplement the on-farm interview survey after the conclusion of on-farm data collection. Three categories of questions were included: (a) key data, where information from additional farms was expected to improve the interpretation of information collected on-farm (these questions were included verbatim); (b) questions that required refinement for online survey delivery (these questions were different from the interviews but captured similar information); and (c) questions that had been omitted from the interview questions to manage interview duration, but where some data were still considered useful to collect, covering animal behaviour, farmer attitudes, meat quality, and detailed data on the cost of fire and insurance. Questions were presented grouped by theme to facilitate survey flow. The survey was delivered using Qualtrics software (version January 2021). The survey is available in full in the Supplementary Materials (S2).

#### 2.2.2. Study Area and Data Collection

The target population for the survey was cattle and sheep farmers whose land had been burnt in the 2019–2020 bushfire season (Figure 1B), who had not participated in the previous research interviews. Inclusion criteria for consenting participants were having at least 10 or more beef cattle or 200 or more sheep on the farm they are involved with and having bushfire on the land of that farm.

The online survey was piloted with two farmers and took approximately 45 min to complete. The survey was then made available to farmers by posting on the Meat and Livestock Australia (MLA) project website, and advertised via media releases, associated print and radio coverage, social media, newsletter advertisements, MLA contacts, district veterinarians, and private veterinarians in fire-affected regions. The survey was available online for 16 weeks, from 2 February to 24 May 2021. In total, 30 respondents consented to participate; however, only 12 met the selection criteria and provided sufficient data for analysis.

### 2.3. Ethics

All aspects of the interview and online survey studies were approved by the University of Melbourne Human Research Ethics Committee (ethics ID 2057148.1 and 20700, respectively). Key researchers (BC, CP, JWW) underwent ‘trauma-informed care’ training prior to conducting interviews. Prior to commencing interview data collection, a plain-language statement was provided to informants that described the research project, approach, what the research was about, the informant’s role, withdrawal, and possible benefits of the research, and all participants signed a consent form. Prior to completing the online survey, participants were required to indicate their informed consent and understanding of a plain-language statement.

### 2.4. Data Analysis

Interview and survey responses were exported as comma-separated text files from Qualtrics, and data cleaning was undertaken in R statistical software v4.1.2 [49] (interviews) or Microsoft Excel (survey, Office 2019 version). Descriptive analyses were performed in R and Microsoft Excel. Observations with numerical values that were biologically implausible were excluded from the calculations of descriptive statistics unless they could be verified. When calculating statistics to describe the outcomes for burnt livestock at both the farm level and also across all farms, only animals reported as burnt were included, excluding those reported as singed. All cattle were considered together for the analysis of burnt animals, rather than separating dairy and beef cattle, as only one property had dairy cattle that were burnt.

Free-text comments from interviewed farmers recorded in each section of the questionnaire were manually reviewed, summarised, and cross-referenced between sections. Free-text comments recorded to describe the lessons learnt by the interviewed farmers were analysed using a modified thematic analysis approach [50]. Specifically, the verbatim comments were reviewed by CP and categorised into preliminary themes and sub-themes. These preliminary themes were then compared and further refined to result in a final list of themes, with each comment attributed to one or more themes.

Likert-scale data from the survey were summarised by categorisation, the calculation of mean response, and qualitative interpretation. Free-text responses from survey participants were categorised and interpreted alongside relevant categorical data where low numbers of responses were received. Where sufficient data were available to justify, free-text fields were analysed using a modified thematic analysis approach. Further statistical analysis was not justified based on the small number of responses and resulting limitations on statistical power and external validity. The results of this study are interpreted as illustrative of the experiences of the responding farmers, rather than as representative of the wider population.

## 3. Results

### 3.1. Participating Farms

A total of 58 farms participated in the study, 46 in face-to-face interviews and 12 in the online survey. Figure 1 shows the geographical distribution of participating farms, overlaid with the extent of the area burnt in the 2019–2020 bushfire event (figure adapted from Cowled et al., 2022) [48]. The farm sizes ranged from 16 Ha to 2950 Ha with a median of 216 Ha. Table 2 summarises the livestock species present on the farm. Almost all participating farms had cattle, with a median of 133 head per property (range: 13 head to 1840 head); 14 farms had sheep with a median of 930 head per property (range: 12 to 12,219). One property had eight goats. Thirteen farms had more than one livestock species on the property. Of the respondents, 11 identified as female and 45 as male, with 2 respondents not indicating their gender. The median age of respondents was 60 years (range 30–79 years). All participating farms experienced fire during the 2019–2020 fire season in South-Eastern Australia, with most experiencing their most severe day of fire on farm between 30 December 2019 and 4 January 2020.

### 3.2. Animal Health

#### 3.2.1. Burnt Livestock

Animals were reported with burn injuries on 26 (45%) of the 58 participating farms (Table 3). Burn injuries were reported in both sheep and cattle, primarily in single species, although four properties had both sheep and cattle burnt.

On properties where livestock were burnt, the number of animals burnt and their outcomes varied substantially. On the nine properties with burnt sheep, a median of 8% of all sheep were burnt (range: 0.3% to 100%). On the 21 properties with burnt cattle, a median of 25% (range: 0.1% to 100%) had burns. The majority of burnt animals either died as a result of their injuries or were euthanised for welfare reasons in the immediate aftermath of the fire, and the proportion of burnt animals that ultimately survived was low for both species (across all farms, 10% of burnt cattle and 2% of burnt sheep survived). There was only one property on which any sheep survived their burns and were not euthanised, with 30 of the 180 burnt sheep (17%) surviving.

Few respondents were able to describe the types or locations of burns in either dying or surviving livestock, and there was no pattern that could be identified to describe how the distribution of burn lesions might be associated with outcome. However, cows and heifers with burnt udders were observed by four respondents to fare poorly in the long term: recovery was prolonged in those that did survive, while others made only a partial recovery and were culled due to a loss of function.

Four interviewees raised the euthanasia of burnt animals as a concern when asked if there was anything else they wanted to share about their experience. There was uncertainty or disagreement about which burnt animals should be euthanised. One participant felt that animals had been euthanised unnecessarily, and another reported that animals initially deemed able to survive were subsequently euthanised when revisited by government representatives. Another respondent reported that a government veterinarian’s assessment that their burnt animals did not require euthanasia gave peace of mind that the livestock were not unreasonably damaged. Finally, one farmer expressed that at the time of the fires, they were uncertain about their ability to use the correct technique to humanely destroy livestock by gunshot. This farmer felt that an increased awareness of resources to support correct technique would be of value.

#### 3.2.2. Other Health Conditions

Burns were not the only condition affecting the health of animals on fire-affected farms. Of the 46 interviewed farmers, 30 reported health issues other than those directly related to burns in their livestock in the 6 months following bushfire. In the online survey, seven farmers reported observations of specific health conditions in cattle, and four farmers reported specific health conditions in sheep. A summary of conditions is shown in Table 4, excluding reproductive diseases, which are discussed in the subsequent section.

Several of the conditions reported in Table 4 resulted in livestock deaths. Twenty of the interviewed farmers reported deaths from health conditions they considered associated with bushfires, including ruminal acidosis, respiratory disease, plant toxicities, and bovine ephemeral fever, in addition to unexplained deaths and misadventure (fire-damaged trees falling on animals). Plant species reported to be associated with livestock toxicities on seven farms included kikuyu (*Cenchrus clandestinus* (Hochst. ex Chiov.) Morrone [51]), lantana (*Lantana camara* L. [51]), bracken fern (*Pteridium esculentum* (G.Forst.) Cockayne [52]), castor oil plant (*Ricinus communis* L. [51]), fireweed (*Senecio madagascariensis* Poir. [51]), and lush clover (*Trifolium* L. [51] species, leading to bloat).

#### 3.2.3. Livestock Management and Biosecurity

Livestock body condition is an indicator of nutritional management across the participating farms. Data to estimate changes in livestock body condition scores (BCS) [53] following the fire were provided by 45 farms for cattle and 11 farms for sheep. Before the fire, most farms reported an average BCS of 2.5 out of 5 or greater in cattle (41/45) and sheep (11/11). An increase in the average BCS of livestock between pre-fire and the time of data collection (interview/survey response) was reported for 33/45 (73%) of farms with cattle and 9/11 (82%) of farms with sheep, with a decreased condition score reported in just 2 farms with cattle and none with sheep (see Table 5).

Although on-farm conditions in the aftermath of fires, such as the mixing of animals due to the loss of fencing, may lead to compromised livestock biosecurity, there were few diseases reported that could be attributed to biosecurity breaches. Reports of the mixing of previously separated groups of livestock are summarised in Table 6. One farm suspected bovine pestivirus was introduced into the herd in the post-fire period, although it was unclear whether this occurred through the mixing of livestock with neighbouring livestock (despite subsequent quarantine) or through new cattle bought onto the property. One further farm experienced confirmed cases of pestivirus following the introduction of new livestock after the fires. Theileriosis was observed in a different farm following new livestock introductions, despite treatment for ticks after arrival. Nine properties reported the length of time they quarantined new livestock for on arrival, with a median time of 7 days (range: 2–61 days).

#### 3.2.4. Reproductive Health

##### Cattle

In total, farmers from 52 farms provided information about the reproductive health of cattle on the property, including all farmers interviewed and six online survey respondents. Interviewed farmers had the opportunity to provide more detailed information regarding reproductive measures, while the online survey only solicited brief subjective observations.

Of the 46 interviewed farmers, 14 reported observing abortions in their cattle since the fires. Most properties reported pre-fire abortion rates of 0 to 1% (13 properties), with one reporting up to 3% being typical in 2017–2019. The mean estimated abortion rate on these farms following the fire was 5.3% (median: 3.0%; range: 0.4–23%). Two farms reported an abortion rate of greater than 10% in their herds following the fire. In addition, two of the six farms completing this section of the online survey (33%) observed an increase in the number of abortions since the fire when compared to the previous three years. However, 35 of the 46 interviewed farmers observed no abortions or abortion occurrence typical of their farms’ history, and four of six farmers completing this section in the online survey observed no substantial increase in abortions following the fires.

Congenital abnormalities in calves were reported on four farms (two each in the interview and online survey cohorts) in conjunction with the increased numbers of abortions. One interviewee suspected that these abnormalities were associated with an incursion of bovine pestivirus infection, while the other suggested that the congenital defects observed in their herd may be genetic rather than fire-related.

Of the 46 interviewed properties, 34 had joined cattle since the fires, and 38 had calved since the fires. Of the farms that had joined since the fires, 18 reported the results of pregnancy diagnosis in sufficient detail for analysis (reported proportion pregnancy-tested-in-calf (PTIC)) and 25 of the farms on which cows had calved since the fires reported valid live-calf percentages. The results for these observations are summarised in Table 7, where a small proportion of farms in both groups observed moderate to major decreases compared to recent, non-fire years. However, there were no substantial changes in reproductive performance reported by respondents to the online survey.

Two interviewees reported suspicion that bull fertility was decreased following the fires, with observations of delayed calving dates despite joining at the usual time of year for their farms. One of these farmers elected to sell and replace the bull, while the other noted that bull fertility appeared to improve over time. One farmer also observed that cows on the property appeared to stop cycling following the fires. Unintentional matings due to a loss of fences were observed on three farms. Another three farms had an extended joining period, two because the bulls could not be segregated from the rest of the herd, and one intentionally because conception rates were lower in drought-affected cows. One farmer reported that a loss of infrastructure in the fire meant that they could not intervene in any reproductive matters but did not elaborate on what specific tasks were not possible.

Other reported issues related to reproductive health in cattle included complications at parturition (dystocia and calving paralysis) noted on five farms, and concerns regarding calf health, either directly related to the udder function of their dams (two farms) or non-specific ill-thrift and death in calves (one farm).

#### Sheep

Six of the farms with sheep reported data for lamb-marking percentages since the fire, with varying effects observed between interview and online respondents. All three of the interviewed farms with sheep that had marked lambs since the fires recorded marking percentages lower than in previous years (pre-fire median lamb marking of 125%, range: 120% to 127%; post-fire median of 106%, range: 105% to 107%). One interviewee specifically observed an increase in the mismothering of twin lambs. By contrast, all three of the online respondents who commented on the marking percentage reported no change in this measure compared to the previous three years. A large increase in reproductive-related issues (abortion, stillbirth, and lamb losses around lambing) associated with an outbreak of campylobacteriosis was reported on one farm in the online survey. The route of entry of the campylobacter infection to the flock was not identified by the respondent, but the farmer noted that flocks were in close proximity in containment pens, which may have facilitated spread. There were no other significant changes noted in reproductive measures on sheep properties.

### 3.3. Farm Recovery

Initial recovery activities related to livestock most commonly included providing water and supplementary feed to the remaining livestock, the repair of fencing, and livestock movement (including the removal of surviving animals from the property, and in the longer term, both selling and buying livestock). Pasture management during recovery in the months following bushfire was also described.

#### 3.3.1. Supplementary Feed and Water

Supplementary feed was an essential part of maintaining the welfare of the animals on the burnt farms. Supplementary feed was provided by the majority of the respondents in both interviews and the online survey (52/58, 90%), with feeding commonly extending for some months following the fires (49/51 (96%), one non-response). Of the 52 farms that provided supplementary feed following the fires, almost half (21, 40%) had not been providing any supplementary feed prior to the fires.

Information on the specific types of supplementary feed provided was sought in both the interviews and the online survey. Twenty-five out of fifty-one farms (49%) fed concentrates (grain or pellets) in addition to roughage (hay, straw, and/or silage), while the remainder fed roughage alone (with one participant not recording the type of supplementary feed used). Of the 19 interviewed farmers who fed concentrates following the fire, 13 (68%) had not been feeding concentrates prior to the fires.

Questions around the provision of water were not asked in a structured way during interviews, but 5 out of 46 participants described this as an important part of their initial recovery. Three key strategies were described, including erecting emergency fencing to contain animals in areas where water was available, using generators to pump water to the locations of livestock, or making emergency repairs to water pipes.

#### 3.3.2. Livestock Movement

In the first week following the fire, several interviewed farms chose to remove livestock from the property, including selling animals to salvage slaughter (7/46 farms), selling animals other than to salvage (5/46 farms), and transporting animals to agistment (4/46 farms) or to another farm block (3/46 farms). These movements reflected decisions made rapidly in the very early stages of recovery.

Details were also gathered about livestock movements in the first six months following the fire to understand non-emergency movements. Some respondents moved animals either to agistment (twelve farms) or to another farm block (seven farms). Eight farms sold livestock with the intent to buy replacement livestock back in later, while eleven farms bought animals to replace those lost or sold in the first 6 months after the fire. Of the eight farms that sold livestock with the intent to buy back in, seven reported the reasons for selling livestock as largely practical or borne out of necessity, rather than because it was the most economically beneficial option. Examples of necessity included to reduce workload or emotional burdens, or due to the loss of infrastructure required for feeding. None of these participants expressed any regret for these decisions when considering lessons learnt or what they might have done differently.

#### 3.3.3. Infrastructure, Animal Handling, and Management Procedures

Online survey participants were specifically asked about the behaviour of their livestock following the fires, and most had observed no change in behaviour when compared to prior to the fire event. However, it is worth noting that one participant observed major behavioural changes in their livestock, commenting that “[the cattle] seem to be frightened and are not as quiet”. In addition, one interview participant noted that animals were “…gate-shy and skittish…” following the fires, and another noted that their cattle were very difficult to move in the face of the fire itself. This suggests that the experience of fire events has the potential to affect animal behaviour and handling.

Farm infrastructure specific to livestock that was lost or damaged on interviewed farms included fences and cattle or sheep yards, with subsequent impacts on animal handling and management. Erecting emergency fencing to secure livestock in the first week following the fire was common (32 of 46 farms interviewed, 70%). Cattle yards were destroyed on 17/46 farms, and sheep yards were rendered unusable on 4/9 farms with sheep, with a total of 19 farms affected. In some cases, farmers had a second set of yards or could reasonably use a neighbour’s yards in the recovery period. The normal timing of animal health management procedures was impacted on 13 of the 19 farms where yards were damaged, with routine husbandry activities (Table 8) being delayed compared to the typical time of year at which they would be performed or skipped entirely. In addition, seven farms reported that due to fire damage to yards, they could not treat animals that were injured or unwell, for example, cattle with mastitis or lameness. One farm specifically reported that they had to destroy additional livestock in the days after the fire, as a loss of their yards meant that they were unable to nurse injured animals, despite having the desire to do so. Labour scarcity due to prioritising recovery activities, such as re-fencing, was also reported to have contributed to the above changes to the normal timing of livestock management activities.

#### 3.3.4. Pasture Recovery

The 2019–2020 bushfire season followed a prolonged period of drought in South-Eastern Australia. Rainfall then followed soon after the fires in the 2019–2020 season in all study regions. Sufficient rainfall to stimulate substantial pasture growth and allow reduced hand-feeding was reported on 27 of 58 farms (47%) by the end of February 2020, with a further 11 farms (19%) receiving this rain by the end of March and the balance of farms by the end of April with the exception of one farm (located in Victoria), which did not have a drought-breaking rainfall event until July 2020. This rainfall following the fires was reported to contribute to damage to topsoil through further erosion on 23 of 58 farms (40%) and to have caused the contamination of water sources on 27 farms (47%). Water contamination in most cases was mild and did not affect animal health due to proactive identification and management (the provision of alternative water sources).

Complete pasture recovery from fire damage was subjectively reported to occur less than one month after the main fire by 14 of the 58 respondents (24%), within 3 months by 23 respondents (40%), and by 3–6 months post-fire by a further 14 of 58 (24%). Recovery took 6 to 12 months on two farms, with a further five farms reporting that their pastures had still not recovered at the time of interview between 9–12 months following the fire.

The interviewed farmers provided additional details about pasture recovery. Over half (24 farms) had identified areas of the farm that would require re-sowing as part of long-term fire recovery due to either pasture death or negative changes to pasture composition. Most felt that substantial areas of the farm needed to be re-sown. The active pasture recovery activities undertaken by the interviewed farmers varied, with the most common presented in Table 9. Sixteen farms did not report undertaking any pasture recovery activities in the first 6 months after the fire. Three interview participants noted that pasture recovery was variable across their farms. Two farmers whose farms were not stocked during early recovery (due to the sale of livestock or agistment at another location) reported excellent pasture recovery outcomes. Some subjective observations of the recovery of distinct pasture species included that native pastures and kikuyu recovered well, while three farms identified that clover had returned poorly. It is noted that the good recovery of kikuyu was associated with toxic effects in cattle grazing the pasture on two farms.

Weeds were a major concern during fire recovery on-farm, being noted on 19 of 58 farms (33%) during pasture recovery, despite this information only being specifically sought during the online survey. Two interviewed farmers specifically mentioned spraying for large amounts of weeds as part of their pasture recovery, and 12 of 46 (26%) spontaneously reported an increase in weeds following the fire. In the online survey, seven out of twelve reported an exacerbation of existing weed species known to be on the farm, and two also reported new species of weeds. The presence of these weeds did not necessarily result in an overall change in pasture composition. Occasionally, these weeds resulted in livestock poisonings (see Section 3.2.2 above). The specific weeds mentioned are summarised in Table 10.

#### 3.3.5. Lessons Learned

Open-ended questions about lessons learned through the experience of the 2019–2020 fires were asked both in interviews and the online survey. Several key themes occurred, as summarised in Table 11. Participants offered specific and actionable advice intended to inform other farmers who may face similar circumstances in the future.

## 4. Discussion

This study presents a descriptive overview of animal health and farm recovery on sheep and beef farms experiencing bushfire in South-Eastern Australia after the 2019–2020 fire season. While similar observations are often made during the post-fire period on individual or a small number of farms, these are rarely collated systematically to provide a broader understanding. The results presented are not intended to be representative of all sheep and cattle farms burnt during the 2019–2020 season but rather to offer useful insights to generate more specific hypotheses that could be investigated in future fire seasons. It is challenging to conduct research in a post-bushfire context where farmers are rightfully focussed on their farm recovery and other personal matters, but we propose that as bushfire risk increases, evidence-based insights are extremely valuable to help support fire prevention and recovery activities.

### 4.1. Burnt Livestock

Almost half of the farms in this study had burnt livestock. This does not reflect the broader frequency of farms with burnt animals in these bushfires (due to many farms being recruited in a case–control design) but rather provides insights into the effects of fire on surviving livestock that have had substantial exposure to fire conditions. However, the frequency of fire injury within a farm was not specified at recruitment into the study, and the proportion of livestock burnt on a single farm varied widely. In these burnt livestock, the overall survival was low, with only 2% of burnt sheep (all on one property) and 10% of burnt cattle ultimately surviving their injuries. Sheep appeared to fare worse than cattle, consistent with the existing literature [54]. The majority of burnt animals either died in the paddock or were euthanised shortly after the fire. Two factors contributing to these low survival rates may be the severity of injuries or the conservative assessment of prognosis by veterinarians, preferring to euthanise rather than risk adverse welfare for burnt animals (described in Cowled et al. 2022) [4]. The available data do not allow the impact of these two factors to be differentiated.

### 4.2. Other Health Conditions

In addition to direct burn injuries, many of the 58 farms reported greater-than-normal rates of disease in their livestock in the months after fire. The most common health issues included intermittent or long-term lameness, plant toxicities, ocular disease (commonly reported by farmers to be ‘pinkeye’, which is primarily caused by Moraxella bovis infection), and respiratory disease. Although over half of the surveyed farms reported increases in disease, the frequency of individual diseases was relatively low. There were no high prevalence diseases that could be identified as requiring specific broad-scale preventive measures post-fire. Given the variation in the relevant prevention strategies for the conditions reported, early detection and treatment on a reactive case-by-case basis is likely to be the most reasonable approach to the management of disease in surviving livestock in the post-fire period. In most cases, general disease prevention should focus on nutrition and optimising livestock management within the constraints of the post-fire context, including the appropriate use of livestock containment areas.

Eye disease was the most commonly reported health issue in cattle following fires in this study, which is consistent with contemporary observations in humans [22]. It is possible that exposure to smoke and heat may damage the cornea, which would increase susceptibility to infection [55]. However, the risk of pinkeye is further increased in dusty environments, such as livestock containment areas, which are recommended in the post-fire period. In addition, hot and dry environmental conditions associated with fires are also conducive to the spread of M. bovis [55]. Although it is possible that exposure to fire and changes in husbandry following fires increased the risk of pinkeye, the incidence of pinkeye in these livestock may have been high, for example, due to drought conditions, regardless of the presence of fire on the farm, and it is difficult to interpret the role of fire specifically. Regardless, close monitoring for and the early treatment of pinkeye in the post-fire period is prudent.

The effective management of livestock containment areas and supplementary feeding is likely to play a significant role in the prevention of a number of health conditions in the post-fire period. Moving livestock to containment areas or ‘sacrifice paddocks’ can help meet urgent livestock welfare requirements (which can be described simply as “feed, water and fencing”), allowing time for boundary fence repair or reconstruction as well as facilitating the recovery of fire-damaged pastures. Containing livestock to smaller areas of the farm can also facilitate the frequent monitoring of burnt livestock while recovering from their injuries and the early detection of other diseases and mortality. The appropriate location, design, and stocking rates of containment areas can reduce the occurrence of health issues such as respiratory or ocular disease and lameness [31,56,57,58]. It would be ideal for these areas to either be established as a preparedness activity well in advance of fire risk (also providing benefits in other weather-related circumstances such as drought), although they can also be rapidly established in the aftermath of fire if resources and labour can be made available, as was the case for one of the survey respondents with a large sheep flock.

Ruminal acidosis, occurring when fermentable carbohydrates are fed in large amounts without gradual introduction or without adequate roughage being fed concurrently, may cause reduced weight gain, diarrhoea, dehydration, and death, as well as secondary laminitis (resulting in lameness) in both sheep and cattle [55,59]. Of the 46 interviewed farms, 10 were identified as being at-risk for ruminal acidosis, defined by two characteristics: animals were not already receiving grain or pelleted feed prior to the fire and did receive grain or pellet supplements post-fire. Eight of these at-risk farms used a pelleted feed and did not report deaths due to acidosis; one farm fed barley and reported no issues, while the remaining farm fed both pellets and varying types of cereal grains to cattle using a new automated feeder and experienced deaths when increasing the grain feeding rate. That farmer reported that they did not have prior experience with the grain feeding of cattle or using an automated feeder and reflected that an increased awareness of this risk and training to mitigate it may have avoided the losses. Another interviewed farmer who reported acidosis described the early weaning of dairy calves onto grain, as usual weaning paddocks had not recovered from burning, with associated cases. One sheep farm in the online survey also reported acidosis, but no further information about the circumstances was elicited. The prevention of ruminal acidosis relies on the gradual introduction of grain or other highly fermentable carbohydrates and the provision of adequate roughage. Further risk mitigation strategies can include the addition of rumen buffers or neutralisers (e.g., sodium bicarbonate or magnesium oxide) or rumen modifiers, such as ionophores, to supplementary feed [60]. The careful management of concentrate (grain) feeding is important in the post-fire period, although introducing concentrates gradually and providing adequate roughage depends on the availability of alternative feeds.

### 4.3. Biosecurity and Weeds

Despite many reports of groups of animals mixing, either on their own property or with neighbouring livestock, there were few instances of infectious disease that could be attributed to biosecurity breaches recorded in this study, although those that did occur led to potentially costly losses. The observed increase in weeds may be driven by three factors, namely, the introduction of new weeds through purchased feed (such as weed-contaminated hay), vehicles accessing paddocks during fire response and spreading weed seeds, or the overgrowth of endemic weeds secondary to damage to pastures and topsoil, which was reported by one third of the interviewees [20,61,62]. Some weeds, such as *Acacia* Mill. [51] species, are also known to be fire-activated, leading to the further proliferation and infestation of paddocks [63]. While the relative importance of these pathways cannot be determined from the data in this study, the impact of weeds in fire recovery was emphasised by many participants across the two studies.

Plant toxicities were the most common cause of death in livestock in the post-fire period in this study. The prevention of plant-associated deaths post-fire is dependent on both grazing management and awareness and the identification of potentially toxic plant species. Grazing management is also important to facilitate pasture recovery, as overgrazed pastures can be slower to recover and favour the growth of weeds due to reduced competition from primary (intended) pasture species. The unrestricted grazing of certain pasture species in the absence of other feed sources can also lead to livestock deaths, such as from kikuyu poisoning or pasture bloat [53,64,65], especially where rainfall post-fire has stimulated rapid post-summer pasture growth. Effective management strategies to minimise the chance of plant toxicities include the provision of hay or other roughage prior to grazing at-risk pastures and strip grazing with electric fences to reduce the toxic dose ingested at a single point in time [55]. Strategically timed communications about common species associated with toxicity may help farmers mitigate these risks, alongside support to ensure that alternative roughage can be made available in the post-fire period.

### 4.4. Livestock Nutrition

Although fire on a property may be expected to negatively impact the nutritional status of livestock, in this study, the opposite was observed. The majority of properties reported increases in BCS in both cattle and sheep following the fires, possibly linked to drought conditions preceding the fires and good rainfall in the months following the fires. On many farms, this likely led to increased pasture availability compared to the months before the fires, alongside increased supplementary feeding following the fires that may have improved the nutritional status of the animals. The increased BCS observed likely contributed to the incidence of dystocia on some farms. Our results suggest that when pasture recovery is aided by good rainfall, and if adequate supplementary feed can be provided in the interim, fire on a property does not necessarily compromise the nutritional status of surviving livestock; however, this may not be true under different post-fire conditions.

### 4.5. Reproductive Health

Although most farms reported no change in reproductive performance in their cattle or sheep following the fires, a small number of farms reported increased rates of abortion, or reduced PTIC or live calf rates in cattle, and reduced lamb-marking percentages in sheep. These data are not sufficient to determine how bushfires affect reproductive function but indicate that further investigation may be valuable. Possible mechanisms for reproductive compromise post-fire include the increased incidence of infectious diseases due to stress and the associated immune compromise, management changes or biosecurity issues [55], the effects of burns on reproductive organs and mammary tissues [32], and the effects of heat or smoke on fertility or foetal development [66,67,68,69,70]. In humans, the exposure of pregnant women to fine particulate matter has been associated with premature birth; however, the effect is small, and smoke inhalation has not been shown to affect foetal development in the few studies in other species [69,70,71,72]. It is therefore unclear whether exposure to smoke in the fires might play a role in the increased abortions seen in cows following the fires. In bulls and rams, heat is known to reduce fertility [67,68] and serving capacity may also be affected by burns to the penis or testes or scar tissue formed during burn healing [32]. Further research would be required to understand both the prevalence of reproductive compromise and the mechanisms by which bushfire exposure might affect reproductive function, and whether there might be a way to mitigate these effects.

### 4.6. Farm Infrastructure and Management

Infrastructure loss due to fire damage affected farmers’ ability to perform routine management procedures such as vaccination, drenching, and lamb marking, to manage grazing using normal strategies, and to treat injured or diseased livestock in the weeks to months following the fire. There are likely to be associated impacts on animal health, welfare, and production, for example, through increased burdens of internal and external parasites, particularly in sheep flocks and young animals, and increased susceptibility to diseases for which vaccines are routinely used [54,73,74]. The delayed castration of calves and lambs could lead to mis-mating, which may have health consequences for animals that become pregnant from these matings, in addition to consequences for the management of these animals. As marking is often the best opportunity to assess the number of surviving young, particularly on sheep properties, delays to marking might also delay the detection of abnormal mortality rates and, consequently, the diagnosis of significant herd-level health problems. The prevention and treatment of disease in the post-fire period may also be constrained by a lack of infrastructure, an important consideration for advising veterinarians. On properties where infrastructure has been lost and access to suitable facilities on neighbouring properties is not practical, the use of temporary yards for undertaking essential husbandry activities may be appropriate.

The recovery activities undertaken by surveyed farmers following the Black Summer bushfires were largely consistent with those described in the existing government and industry literature [14,20,25,27,39,43,44]. Post-fire pasture recovery activities in active use have not been well described, and several strategies were reported in this study. The further exploration of the effectiveness and relative cost of different pasture recovery strategies specific to grazing land, as well as the consideration of how different post-fire pasture recovery needs to be from the routine management of pastures with low feed availability (such as in drought), may be of value. Agronomists were the most common type of professional consulted in the period following the fire (see Supplementary Materials (S3)), suggesting that farmers identified the value of assistance with pasture recovery and/or advice on cropping. Based on farmers’ responses to the open-ended questions about lessons learned from the Black Summer bushfires, the management and use of water are also important, particularly in preparation for fire events. Recovery activities related to water may also be more common than indicated by this study and should be specifically included in future research.

### 4.7. Limitations

While this study provides an overview of observed diseases increasing morbidity and mortality in Australian cattle and sheep following bushfires, several limitations should be considered. It is not possible to definitively separate disease occurring in association with bushfires from disease that would have occurred regardless. Disease occurrence is variable between years, depending on several factors, including seasonal conditions, compounded by the lack of baseline data on the incidence of endemic diseases on Australian beef and sheep farms. Most farms studied had experienced significant drought prior to the 2019/2020 bushfires, and the observations reported here may have been influenced by the effects of drought in addition to bushfire. The Black Summer bushfires were an unusually severe fire event with unusual fire behaviour, so it may not be appropriate to extrapolate these findings to other fire seasons or events. Further, the studied farms are not expected to be representative of all cattle and sheep farms affected in 2019–2020, with these data presenting insights rather than a comprehensive overview. Selection biases influencing participation include an increased likelihood of participation by farmers with strong opinions about fire preparedness, response, and recovery and those who had the emotional resources to agree to participate (therefore excluding some farmers with severe trauma or mental health effects). In addition, while almost all fire-affected farms from NSW were included in the sampling frame for the study, the selection of farms for inclusion in Victoria and through the online survey was non-random, which may have introduced selection bias. Generalisability is further limited because the majority of the interviewed farms were beef cattle farms, and the type and incidence of disease might differ between species and production systems. There would be value in a similar study more focussed on sheep production systems in future years, where sheep farmers are substantially fire-impacted, to identify key potential health problems in sheep. Additionally, there is uncertainty surrounding the accuracy of the diagnoses reported, being based on farmer observations rather than strictly diagnosed by animal health professionals or confirmed by laboratory testing, and the reported numbers of the affected animals are estimates provided by the farmers. Therefore, this study provides an overview of the health conditions and recovery activities occurring in farms following fires but does not establish the true prevalence or impact of conditions that occur.

## 5. Conclusions

The results of this study illustrate that bushfire events have a broad range of health and welfare consequences for livestock, often associated with farm management during the recovery period rather than fire injury or direct fire impacts on animals. The specific challenges faced by individual farms may vary substantially. Therefore, general advice that may improve the underlying management factors contributing to disease occurrence rather than specific disease-prevention advice appears most appropriate, in addition to treatment on a case-by-case basis. Understanding the competing priorities and logistical challenges for farmers during recovery, and recognising the insights given by these farmers about recovery and lessons learnt, will help support farmer wellbeing, farm recovery, and the management of risks to animal welfare.

## Figures and Tables

**Table 1:** Summary of the hypothesised biological mechanisms leading to potential impacts on livestock health in the aftermath of bushfire.

Nutritional stress due to loss of feed, leading to increased disease and reproductive and production losses [21,22,23].
Use of feed to which animals are unaccustomed, leading to diseases such as grain overload, enterotoxaemia, and pasture toxicities [12,24,25,26,27].
Greater access to toxic weeds due to their introduction in contaminated feed or overgrowth in fire-damaged pastures [24,28].
Nutritional and other stressors may increase the effects of existing gastrointestinal parasite burdens [27].
Nutritional and other stressors, as well as changes in management associated with fire, may lead to the increased rates and severity of infectious diseases, for example, changes to the infection pressure of endemic diseases as a result of the increased shedding of infectious agents by animals under stress, post-fire management strategies (such as containment feeding), and the effects of fire on the environmental burden of infectious agents [12,27,29,30,31]; biosecurity issues, such as loss of fencing, facilitating the introduction of novel infections into a herd/flock or the abnormal spread of infections within a herd/flock [27,29]; stress and co-mingling of animals due to evacuation or other livestock movement may predispose animals to respiratory disease such as pneumonia [19].
Wounds associated with exposure to fire may increase susceptibility to flystrike, especially in sheep [6,32].
Contamination of water sources by the carcasses of animals that died during fires may lead to disease, for example, through water contamination with botulinum toxin from infected carcasses [33].
Loss of infrastructure, such as yards, may affect farmers’ ability to treat sick animals or perform routine management activities [27].

**Table 2:** Livestock species kept on 58 fire-affected farms in South-Eastern Australia participating in a study of farmers’ observations of livestock health and welfare, biosecurity, farm impacts, and recovery in the months following bushfires in 2019–2020.

Species	Number of Farms			Number of Animals	
	Interview	Online Survey	Total	Per Farm Median (Min, Max)	Total Count
Cattle ^1^	46	10 ^^^	57	133 (13, 1840)	15,277
Sheep	9 ^#^	5 ^^^	14	930 (12, 12,219)	40,150
Goats ^2^	0	1 ^^^	1	8 (8, 8)	8

^1^ beef and dairy cattle; ^2^ non-dairy goats. # of interview participants on all farms with sheep also stocked beef cattle. ^ of the online participants, three farms stocked both beef cattle and sheep; one farm stocked beef cattle and goats.

**Table 3:** Number of livestock burnt (percentage of livestock on farm) and their outcomes on 58 fire-affected farms in South-Eastern Australia participating in a study of farmers’ observations in the months following bushfires in 2019–2020.

	Cattle Count (%)	Sheep Count (%)
Number of farms with burnt livestock	21 (38%)	9 (64%)
Total livestock burnt (all farms)	1191 (8%)	1903 (5%)
Outcomes for burnt livestock		
Died in paddock	651 (55%)	868 (46%)
Destroyed for welfare reasons	236 (20%)	964 (51%)
Died of complications	77 (6%)	36 (2%)
Survived but euthanised later	166 (14%)	5 (0.3%)
Survived with full recovery	118 (10%)	30 (2%)
Carcasses unable to be found	20 (2%)	0

**Table 4:** Reported occurrences of animal health conditions in cattle and sheep in the months following bushfires in 2019–2020 on 58 bushfire-affected farms in South-Eastern Australia. Reported as the number of farms reporting conditions (percentage of farms). NA = not applicable to circumstances.

Animal Health Issues	Cattle Count (%)	Sheep Count (%)
Ruminal acidosis	2 (4%)	1 (8%)
Eye diseases	8 (15%)	0
Lameness ^1^	7 (14%)	1 (8%)
Respiratory disease ^2^	4 (8%)	2 (15%)
Plant toxicities	6 (11%)	1 (8%)
Mastitis	1 (2%)	1 (8%)
Bovine ephemeral fever	1 (2%)	NA
Bovine pestivirus	1 (2%)	NA
Misadventure ^3^	1 (2%)	1 (8%)
Unexplained deaths in adult animals	5 (10%)	1 (8%)

^1^ reports of lameness include ‘sore feet’ that may be attributable to injury from walking on hot ground or laminitis; ^2^ reports of respiratory disease include coughing, breathing difficulties, and pneumonia; ^3^ misadventure includes accidents such as fire-damaged trees falling on animals.

**Table 5:** Reported change in body condition score (BCS comparing BCS before fire with BCS 9–15 months after fire) for cattle and sheep on farms in South-Eastern Australia that were fire-affected in the 2019–2020 bushfire season, reported as the number of farms (percentage of farms). Body condition scores were estimated on a scale of 1–5 [53].

Species	Reported Change in Average Body Condition Score Since Fire			
	Decrease	No Change	Increase < 1 Score Point	Increase ≥ 1 Score Point
Cattle (*n* = 45)	2 (4%)	11 (24%)	14 (31%)	19 (42%)
Sheep (*n* = 11)	0	2 (18%)	4 (36%)	5 (45%)

**Table 6:** Biosecurity risk events and mitigation strategies reported on fire-affected farms in NSW and Victoria, Australia, during and following the 2019–2020 bushfire season, reported as the number of farms (percentage of farms). NA = not applicable to circumstances.

	Number of Farms (%)	Biosecurity Mitigation Strategies Used		
		Animal Health Statement	Anthelmintic Drench	Quarantine of Livestock
Livestock groups mixed within a property ^1^	29 (50%)	NA	NA	0 (0%)
Livestock mixed with neighbour’s animals ^2^	27 (47%)	NA	NA	1 (4%)
Purchased new livestock ^3^	14 (24%)	6 (43%)	9 (64%)	11 (79%)

^1^ livestock mixed with other groups of livestock on the same property from which they were usually separate; ^2^ livestock mixed with livestock from neighbouring properties, for example, due to damaged boundary fences; ^3^ new livestock were introduced onto the farm in the months following the fires.

**Table 7:** Reproductive outcomes in cattle reported as the number of farms (percentage of farms) for 29 farms in South-East Australia that were fire-affected in the 2019–2020 bushfire season. ‘Moderate to major’ indicates a change of ≥5.0 percentage points; ‘minor’ indicates a change of <5.0 percentage points.

Reproductive Measure	Change in Outcome Compared to Prior Three Years				
	Moderate to Major Decrease	Minor Decrease	No Change	Minor Increase	Moderate to Major Increase
PTIC * % (*n* = 18)	3 (17%)	3 (17%)	12 (67%)	0 (0%)	0 (0%)
Live calf % (*n* = 25)	3 (12%)	6 (24%)	13 (52%)	3 (12%)	0 (0%)

* PTIC = pregnancy-tested-in-calf.

**Table 8:** Routine husbandry procedures not performed during recovery from bushfires due to fire impacts (such as a loss of handling facilities) on 19 farms in South-East Australia that were fire-affected in the 2019–2020 bushfire season.

Husbandry Activity Delayed or Not Completed Due to Damaged Facilities	Number of Farms (Count)
Re-drafting for grazing management	7
Vaccination or drenching	5
Lamb marking	4
Sales or loading	2
Milking	1

**Table 9:** Pasture recovery activities undertaken in the months following the fire on 46 interviewed farms in South-Eastern Australia that were fire-affected in the 2019–2020 bushfire season.

Pasture Recovery Activities	Number of Farms (Count)
Nitrogen application	14
Sowing fodder crops	12
Superphosphate application	3
Lime application	2
Sowing perennial pasture species	2
Keeping livestock out of affected paddocks for several months	1
Fertiliser application by helicopter	1
Using silage to stabilise soils against erosion	1

**Table 10:** Summary of weeds reported as newly established or increased abundance during bushfire recovery on 58 farms in South-Eastern Australia that were fire-affected in the 2019–2020 bushfire season. Weed types are given as common names reported by farmers and as scientific names at the genus level [51].

Common Name as Reported	Scientific Name (Genus)	Count of Farms Reporting
African daisy, fireweed	*Senecio* L.	9
Thistle	*Carthamus* L.; *Onopordum* L.	5
Wattle	*Acacia* Mill.	4
Inkweed, dyeberry	*Phytolacca* L.	4
Capeweed	*Arctotheca* J.C. Wendl.	3
Blackberry	*Rubus* L.	2
Tobacco bush, wild tobacco	*Solanum* L.	2
Salvation Jane, Paterson’s curse	*Echium* L.	2
Bathurst burr	*Xanthium* L.	1
Bracken	*Pteridium* Gled. Ex Scop.	1
Cape tulip	*Moraea* Mill.	1
Fleabane	*Conyza* Less.	1
Lantana	*Lantana* L.	1
Pine (new shoots)	*Pinus* L.	1
Soursop	*Oxalis* L.	1
St John’s wort	*Hypericum* L.	1
Stinking Roger	*Tagetes* L.	1

**Table 11:** Key themes and specific advice describing ‘lessons learned’ by fire-affected livestock farmers in South-Eastern Australia, as reported in on-farm interviews in 2020 (N = 46).

Theme	Advice	Number of Comments
water	total comments about water	**20**
	increase water reserves (e.g., dams, bores, tanks)	8
	rooftop bushfire sprinkler system (get one or improve existing)	5
	watering/sprinklers (use them, especially around the house)	5
	sprinkler for livestock (use it)	1
	water system resilient to heat (rebuild so it will not melt, e.g., pipes buried)	1
firefighting	total comments about firefighting	**11**
	obtain new or improved firefighting unit (e.g., slip-on *) or hose	7
	prioritise uninsured infrastructure when defending	1
	stay on farm rather than joining fire crew at the expense of defending own farm	1
	obtain firefighting experience	1
	be aware of your own health—smoke exposure and exhaustion	1
preparation	total comments about preparation	**13**
	rebuild with fire in mind	2
	move valuables off-site or to a safe location	2
	prepare early and adequately	2
	do not underestimate what fire can do/size of fire	2
	be self-sufficient in fire plan rather than relying on government/local fire service which may not be available	2
	digitise records	1
	set dates for preparation each year	1
	be aware that roadblocks could be in place for extended time periods	1
power	total comments about power	**11**
	new generator installed since fire	6
	be prepared to lose mains power	2
	change to diesel generators	2
	have extra fuel for generators	1
hazard reduction	appropriate hazard reduction in local bush is essential	**9**
moving livestock	total comments about moving livestock	**11**
	move livestock to bare paddocks, strategic refuge paddocks, or yards	6
	move livestock early	1
	ploughed firebreaks can protect livestock	1
	plan to destock before fire	1
	open gates to paddocks, allowing livestock to move themselves to safer areas	1
	significantly de-stocking after fires allows appropriate care for the remaining livestock	1
communications	obtain or improve access to UHF radios, can also consider satellite internet or aerial booster for telephone	**5**
mental health & social aspects	total comments about mental health and social aspects	**5**
	consider family members’ location	2
	awareness of emotional/mental impact	2
	understand that fire response and recovery requires day-to-day decision-making	1
insurance	ensure insurance is adequate (acknowledging that not everything can/should be insured)	**5**
vegetation	total comments about vegetation	**4**
	reduce vegetation near structures	3
	clear firebreaks around fence lines	1
livestock feed	total comments about livestock feed	**2**
	establish ‘sister farm’ for agistment in case of fire	1
	buy and receive feed ahead of time, knowing roads will be closed	1
other	total comments about other advice	**5**
	build fire-proof infrastructure (steel yards and fencing)	2
	adjoining crops saved paddocks that otherwise would have burnt	1
	take advantage of offers of help early in the days after fire (e.g., offers of agistment)	1
	improve understanding of emergency procedures with livestock (e.g., emergency feeding)	1

* a ‘slip-on’ is a firefighting unit consisting of a tank, pump, and hose, which can be placed in the tray of a ute (pickup truck) for easy transport to the location where firefighting is required.

## Data Availability

The data presented in this study are available on request from the corresponding author. The data are not publicly available due to ethical restrictions.

## Supplementary Materials

The following supporting information can be downloaded at: https://www.mdpi.com/article/10.3390/ani15121764/s1, S1: Bushfire_interviewquestions; S2: Bushfire_onlinesurvey; S3: Bushfire_farmadvisors.

## Abbreviations

The following abbreviations are used in this manuscript:

BCS	Body Condition Score
LLS	Local Land Services
MLA	Meat and Livestock Australia
NSW	New South Wales
PTIC	Pregnancy-tested-in-calf
