# Supplementary material for: Livestock Farm Recovery Following Bushfire in South-Eastern Australia: Impacts on Cattle and Sheep Health and Management
Source: Animals (Basel). 2025 Jun 14;15(12):1764. doi: 10.3390/ani15121764 (PMC12189280; doi:10.3390/ani15121764)
Supplement: Supplementary file 1 [file animals-15-01764-s001.zip › S1_Bushfire_interviewquestions.pdf]

Livestock and Australian Bushfires: Affected Farmer Interviews (concurrent with on-farm sampling)

Start of Block: 0 Pre-interview checks

Q0.1 Has the Consent Form "Project: Health, welfare and biosecurity of livestock exposed to Australian bushfires: an on-farm case control study" been signed by the participant?
(If not, please get Consent Form signed prior to commencing interview)

- Yes (1)
- No (2)

Q0.2 What is the project unique farm identifier for this farm?

For NSW use the LLS Holding Reference Number (HRN)
For VIC identifier should be in the format: Surname_First6numbersofcontactphone (e.g. “Cowled_042085”)

Q0.3 What are the longitude and latitude of the yards where the samples are being taken today?
(If yards not on fire-affected block, take coordinates of house or another landmark that will be visible on google maps. Include as many decimal places as provided by the device used.)

	List details (1)
Longitude (e.g. 150) (1)	
Latitude (e.g. -40) (2)	
Landmark type (if not cattle yards) (3)	

Q0.4 Please read the following text to the participant:

"We're going to talk in detail about the fire on your property in this interview. You are allowed to take a break anytime, if you would like a break please interrupt me and say so. We can finish the interview early at any time for any reason and you don't have to tell me why. If you would like to stop at any point please interrupt me and say so. There are no negative consequences for you if we do not complete the whole interview. Do you have any questions before we begin?"

End of Block: 0 Pre-interview checks

Start of Block: 1 Bushfire presence or absence

Q1.0 Please read the following text to the participant:
"There are nine sections in this interview, some will be quite quick and some may take a bit longer. We'll start with the first section, about details of the fire that occurred on the property we are talking about today."
Q1.1a Do you own the property we are discussing today?

- Yes (1)
- No (2)

Q1.1b Given you are not the property owner, what role do you have on the this property?

- Manager (1)
- Share-farmer (2)
- Other (specify below) (3)

Q1.1c What other role do you have on this property?

____

Q1.1d Given you are not the property owner, do you own any of the stock on this property?

- Yes (1)
- No (2)

Q1.1e What percentage of the stock on this property do you own?

____

Q1.t2 The rest of the questions on this page relate to the 2019-20 fire season. In the 2019-20 fire season:

Q1.2 (In the 2019-20 fire season) Was there bushfire on your farm land?
Bushfire is defined as an uncontrolled fire that occurs in forest, scrub, woodland, grassland or pasture.

- Yes (1)
- No (2)

Q1.3 (In the 2019-20 fire season) For how many days did bushfire burn on your farm?

____

Q1.4a What date did the fire start burning on your property, on the first day of bushfire (in the 2019-20 fire season)? (enter as dd/mm/yyyy)

____

Q1.4b What time on that date, to the nearest hour, did the fire start burning on your property, on the first day of bushfire (in the 2019-20 fire season)?

____

Q1.5 What date was your 'worst fire day' (in the 2019-20 fire season)? (enter as dd/mm/yyyy)

For example, the worst day for fire on your farm, or the day the fire was a highest risk of damaging your farm. This should be the same day used when preparing the farm maps. This day is referred the 'study day' for the rest of the interview and will be the main focus for most of the survey.

____

Q1.6 In what hour on that 'study day' did the fire start burning (or flare up if your property was already burning)?

____

Q1.7a What were the main reason(s) fire ignited on (or nearest) your farm on the 'study day'? Can select one or multiple reasons.

- Main fire front reached farm (1)
- Spot fires (2)
- Persistent embers (3)
- Backburning (4)
- Lightning strike (5)
- An existing fire flared up (6)
- Other (specify below) (7)

Q1.7b What was the other reason the fire ignited on the 'study day'?

____

End of Block: 1 Bushfire presence or absence

Start of Block: 2 Fire severity or intensity

Q2.0 All questions on this page relate to the 'study day' identified previously.

Q2.1 On the 'study day', did areas of pasture burn?

- Yes (1)
- No (2)

Q2.2 What is your estimate of how fast the MAIN fire moved on pasture?

- Fast (>5 km/hr) (1)
- Medium (>1 to (2)
- Slow ((3)

Q2.3 What is your estimate of the height of the flame length on pasture (in metres)?

____

Q2.4 On the 'study day', did wooded areas of the farm burn?

- Yes (1)
- No (2)

Q2.5 What is your estimate of how fast the MAIN fire moved in wooded areas?

- Fast (>5 km/hr) (1)
- Medium (>1 to (2)
- Slow ((3)

Q2.6 What is your estimate of the height of the flame length in wooded areas (in metres)?

____

Q2.7 What is your estimate of the average depth of litter (fine fuel) on the ground in wooded areas (in cm)?

____

Q2.8 How wide was the MAIN fire front?

- Spot fires or narrower than a paddock (i.e.   (1)
- Broad front at least a paddock wide (i.e. >400m) (2)

End of Block: 2 Fire severity or intensity

Start of Block: 3 Fire history, topography and weather conditions

Q3.1 What year did you start on this farm?
(i.e. what year do you have detailed knowledge of the fire history of the property from?)

____

Q3.2 To the best of your knowledge, in what year was the last bushfire on this farm prior to October 2019? (If not known, leave blank)

____

Q3.3a From your observations, did the animals seek particular areas or topography on the farm in the face of the fire?

- Yes (1)
- No (2)

Q3.3b What were the environmental features of the area that animals sought in the face of the fire?

____

____

Q3.4 Think about areas of pasture that were near the fire but did not burn. Overall from your observations, what were the main common environmental features of those areas of pasture?

____

____

Q3.5a
Think about the wind direction on the study day. Was the wind in a single direction or multiple directions?

- Single wind direction (1)
- Multiple wind directions (2)

Q3.5b What direction was the wind blowing from on the 'study day'?

____

Q3.5c
Consider the 12 hours preceding the fire and the rest of the day on that 'study day'. What were the main directions the wind blew from on the 'study day', and what time did they change?

____

____

End of Block: 3 Fire history, topography and weather conditions

---

Start of Block: 4 Farm type and management

Q4.0 Please read the following text to the participant:
"That's the first section complete. Do you want to take a short break, or is it ok for us to keep going?"
Once participant indicates it's ok to keep going:
"The next section of the interview is about details of the livestock on your farm and how you

manage them. The questions relate to the stock you had on farm on the 'study day', just for the main block that was burnt."

Q4.1 Which types of production animals did you have on the block?
- Beef cattle (1)
- Dairy cattle (2)
- Sheep (3)
- Goats (4)
- Alpacas (5)

Q4.2a What type of beef enterprise did you have? (can select multiple)
- Self-replacing commercial (1)
- Self-replacing seedstock (2)
- Trading (3)
- Backgrounding (4)
- Agistment (5)
- Other (specify) (6)

Q4.2b What was the other type of beef enterprise?

____

Q4.2c What type of dairy enterprise did you have?
- Seasonal calving (1)
- Split calving (2)
- Year-round calving (3)

Q4.2d What type of sheep enterprise did you have?
- Self-replacing merino (1)
- Self-replacing prime lamb (2)
- Terminal sire to merino (breeding first cross ewes or terminal lambs) (3)
- Terminal sire to first cross ewes (4)
- Stud (5)
- All wethers (6)
- Trading e.g. trade lambs (7)
- Other (8)

Q4.2e What was the other type of sheep enterprise?

____

Q4.3a How many cattle did you have in each of the following groups on the 'study day' on this block?

	Number of animals (1)
Unweaned calves (1)	
Weaned calves (up to 12 months) (2)	
Yearlings & heifers (12 months to 2 years) (3)	
Adult beef cows (>2 years) (4)	
Adult dairy cows (>2 years) (5)	
Adult bulls (>2 years) (6)	

Q4.3b How many sheep did you have in each of the following groups on the 'study day' on this block?

	Number of animals (1)
Unweaned lambs (1)	
Weaners (up to 12 months) (2)	
Hoggets & ewe lambs (12 months to 2 years) (3)	
Adult ewes (>2 years) (4)	
Adult rams (>2 years) (5)	
Adult wethers (>2 years) (6)	

Q4.3c How many of these other species did you have in each of the following groups on the 'study day' on this block?

	Number of animals (1)
Alpacas (total) (1)	
Dairy goats (total) (2)	
Non-dairy goats (total) (3)	

Q4.4 What is the typical weight of a dry (non-lactating) animal of each of the following types on your farm?

	Typical weight (kg) (1)
Dry beef cow (or steer) (1)	
Dry dairy cow (2)	
Dry non-pregnant ewe (or wether) (3)	

Q4.5 What is the area of the block for each of the following purposes?

	Land area	Units (ha preferred)
	Enter area (1)	
Total area (1)		▼ Hectares (preferred) (1 ... Acres (2)
Area grazing land (includes fodder crops) (2)		▼ Hectares (preferred) (1 ... Acres (2)
Area with commercial crops (3)		▼ Hectares (preferred) (1 ... Acres (2)
Area for other purposes (not usable for livestock) (4)		▼ Hectares (preferred) (1 ... Acres (2)

Q4.6a How did you graze your stock in spring and summer 2019, prior to the fire in your region?

- Set stocking (1)
- Rotational grazing (2)
- Both set stocking and rotational grazing (3)

Q4.6b What months was each grazing approach used?

	Month(s) (1)
Set stocking (1)	
Rotational grazing (2)	

Q4.7 Consider the typical stocking rate on your farm and the landscape of this block. Overall, is your stocking rate:

- High (1)
- Medium (2)
- Conservative (3)

Q4.8a Do you irrigate pasture on this block?

- Yes (1)
- No (2)

Q4.8b What proportion and time of year is the block irrigated?

	Proportion of pasture irrigated (1)	Months of year when irrigation occurs (2)
For this block: (1)		

Q4.9 What was the average body condition score of your animals on the 'study day' and now? (Use the reference charts provided as guidance, score from 1 to 5. Estimate to nearest 0.5 of a condition score)

	Average body condition score on 'study day' (1)	Average body condition score now (2)
Adult beef cows (1)		
Typical beef cattle (2)		
Ewes (3)		
Typical sheep (4)		

Q11.1a Were your stock receiving supplementary feed in spring and summer 2019, prior to the 'study day'?

- Yes (1)
- No (2)

Q11.1b Which of these supplementary feeds were provided to stock in spring and summer 2019, prior to the 'study day'?

- Grain (1)
- Pellet (2)
- Hay/Roughage (includes silage) (3)
- Other (4)

End of Block: 4 Farm type and management

Start of Block: 5 Fire preparation

Q5.0 Please read the following text to the participant:
"That's the end of that section. The next section of the interview is about what you did to prepare before the fire on the study day. Any time you need a break, please interrupt me and we can take a break."

Q5.1 Do you routinely remove the following types of vegetation for fire preparedness: (remove includes clearing, thinning, weed control, etc.)

Large trees (1)	▼ Yes, routinely removed (1) ... No routine removal (2)
Smaller weeds and/or leaf litter (2)	▼ Yes, routinely removed (1) ... No routine removal (2)

Q5.2 Did you remove vegetation between August 2019 and the 2019-20 fires?

- Yes (1)
- No (2)

Q5.3 Did you purposefully graze down refuge paddocks to shelter stock in advance of the fire?

- Yes (1)
- No (2)

Q5.4a Did you have any firebreaks installed prior to the fire on the 'study day'?

- Yes (1)
- No (2)

Q5.4b What kind of firebreaks were installed?

- Temporary firebreaks (1)
- Permanent firebreaks (2)

Q5.5 Did you have access to large volumes of water suitable for firefighting?

- Yes (1)
- No (2)

Q5.6 Did you have a fire plan in place at the time of the fires?

- Yes (1)
- No (2)

Q5.7 Did you plan on staying or going in the event of a fire in 2019/20? Was this what you did?

	Stay on-farm (1)	Go (2)
Planned action (1)	●	●
Actual action (2)	●	●

Q5.8a Did you have firefighting units on the farm? A unit is a mobile water tank, firefighting pump and hose.

- Yes (1)
- No (2)

Q5.8b How many firefighting units did you have?

____

Q5.9a Did you move stock between paddocks on-farm in advance of the fire or in response to the fire? (i.e. to protect them)

- Yes (1)
- No (2)

Q5.9b Which paddock(s) did you move stock out of? (paddock name as listed on your paddock map)

____

Q5.9c Which paddock(s) did you move stock into? (paddock name as listed on your paddock map)

____

Q5.10 Did you move stock off-farm as part of fire preparation?

- Yes (1)
- No (2)

Q5.11 In general, do you rely on the RFS/CFA for fire response?

- Yes (1)
- No (2)

Q5.12 Are there any other aspects of your fire preparations that you think are important that we haven't discussed yet?

____

____

End of Block: 5 Fire preparation

Start of Block: 6 Fire response

Q6.0 Please read the following text to the participant:
"That's the end of that section. The next section of the interview is about what you did to respond on that worse fire day that we're calling the 'study day'."

Check in if the participant needs a break as required.

Q6.1 Did you stay and defend the farm during the fire?

- Yes (1)
- No (2)

Q6.2 How many farm firefighting personnel were available during the fire (on average across the day)?

- One (1) person only (1)
- 2-3 people (2)
- 4-5 people (3)
- 6 or more people (4)

Q6.3a Did farm firefighting personnel fire have personal protective equipment? (for example, face masks, goggles, fire resistant clothing)

- Yes (1)
- No (2)

Q6.3b What personal protective equipment did those people have?

____

____

Q6.4 How many farm firefighting personnel had firefighting training and/or experience?

- All (1)
- Some (2)
- None (3)

Q6.5 Did the farm firefighting personnel have communications equipment (such as radios)?

- Yes (1)
- No (2)

Q6.6 Did you receive assistance from RFS/CFA?

- Yes (1)
- No (2)

Q6.7 Was the fire accessible to firefighting (i.e. safe to approach) on the 'study day'?

- Yes (1)
- No (2)

Q6.8 Did you or the RFS/CFA make active efforts to fight the fire when your property was in fire?

- Yes (1)
- No (2)

Q6.9 Were your firefighting efforts directed towards:

	Yes (1)	No (2)
House (1)		
Other infrastructure (2)		
Paddocks (3)		

Q6.10a Which (if any) of the following activities were undertaken immediately before (in the two weeks before) or on the 'study day'?

- Bulldozed/ploughed fire breaks in advance of the fire (1)
- Back burning during the fire (2)
- Attacking fire with water (ground-based or aerial) (3)
- Blacking out containment lines at the edge of an active fire (with fire, chainsaws etc.) (4)
- Removal of ground fuel (e.g. leaves) (5)
- Targeted watering (6)
- Filled up all firefighting units (7)
- Cut fences (8)
- Other (specify in subsequent question) (9)
- None of these actions taken (10)

Q6.10b For the targeted watering, was this:

- Just around house (1)
- House and other places/areas on farm (2)
- Just other places/areas on farm (3)

Q6.10b For cut fences, which paddocks were those fences between? (please list as paddock pairs or paddock name/laneway, using paddock names as listed on your paddock map)

____

Q6.10c What other preparation actions were taken in the two weeks before the 'study day'?

____

____

Q6.11a Did you lose any of the following farm infrastructure:

- House (1)
- Sheds (includes shearing shed if relevant) (2)
- Equipment/Machinery (3)
- Cattle yards (4)
- Sheep yards (5)
- Fences (6)
- Stored feed (7)
- Windbreaks (8)
- Other (specify in subsequent question, includes water tanks) (9)
- No infrastructure lost (10)

Q6.11b Concerning fences lost, were these boundary or internal fences?

- Just boundary (1)
- Just internal (2)
- Both boundary and internal (3)

Q6.11c What proportion (percentage) of your total boundary fencing was lost?

____

Q6.11d What proportion (percentage) of your total internal fencing was lost?

____

Q6.10e What type of stored feed was lost?

____

Q6.11f What other infrastructure was lost?

____

____

Q6.12 Are there any other aspects of your fire response that you think are important that we haven't discussed yet?

____

____

End of Block: 6 Fire response

Start of Block: 7 Fire recovery

Q7.0 Please read the following text to the participant:
"That's the end of that section. The next section of the interview is about what you did to recover after the study day."

Check in if the participant needs a break as required.

Q7.1a Within the first week after the 'study day', which of the following activities did you undertake?

- Providing supplementary feed (1)
- Sold animals to salvage slaughter (2)
- Sold animals other than to salvage slaughter (e.g. to saleyards or to other producers) (3)
- Undertook activities to secure stock (e.g. emergency fencing) (4)
- Transported animals for agistment (5)
- Transported animals to another farm block (other than agistment) (6)
- Other (specify in subsequent question) (7)
- None of these activities undertaken (8)

Q7.1b In the first week, to which animals and by what method did you provide supplementary feed?

	Provided supplementary feed		How feed supplied	
	Yes (1)	No (2)	In paddock (1)	In feedlot/containment type area (2)
Beef cattle (1)				
Dairy cattle (2)				
Sheep (3)				

Q7.1c What other activities (related to your livestock enterprise) did you undertake in the first week after the 'study day'?

____

____

Q7.2a After the first week and up to 6 months after the 'study day', which of the following activities related to livestock did you undertake?

- Providing supplementary feed (1)
- Transported animals for agistment (5)
- Transported animals to another farm block (other than agistment) (6)
- Sell animals with intention to buy stock back in later (4)
- Bought animals in to replace those lost or sold (3)
- Other livestock-related activity (specify in subsequent question) (7)
- None of these activities undertaken (8)

Q7.2b After the first week and up to 6 months later, to which animals and by what method did you provide supplementary feed after the first week?

	Provided supplementary feed		How feed supplied	
	Yes (1)	No (2)	In paddock (1)	In feedlot/containment

				type area (2)
Beef cattle (1)				
Dairy cattle (2)				
Sheep (3)				

Q11.2a Which of these supplementary feeds were provided to stock after the 'study day' (including up to 6 months later)?

- Grain (1)
- Pellet (2)
- Hay/Roughage (includes silage) (3)
- Other (list below) (4)

Q11.2b For the grain, what type of grain?

- Wheat (1)
- Barley (2)
- Oats (3)
- Lupins (4)
- Other (list below) (5)

Q11.2c For the roughage, what type of roughage?

- Hay (1)
- Silage (2)
- Straw (3)
- Other (list below) (4)

Q11.2d What was the other type(s) of feed/(grain)/(roughage)

____

Q7.2c For the animals sold with intention to buy stock back in later, what was the reason behind this decision?

- Decision based on economics/budgeting (1)
- Feed could not be sourced (regardless of price) (2)
- Not prepared to feed stock (regardless of price) (3)
- Other reason or more detail (specify in subsequent question) (4)

Q7.2d What was the other reason stock sold with the intention to buy back in later?

____

____

Q7.3a After the first week and up to 6 months after the 'study day', which of the following activities related to pasture recovery did you undertake?

- Nitrogen application (once rain came with intent to grow more feed) (1)
- Oversow with annual pasture (2)
- Sow fodder crop (3)
- Other pasture recovery activity (specify in subsequent question) (4)
- No pasture recovery activity taken (5)

Q7.3b What was the other pasture recovery activity undertaken?

____

____

Q7.4a Will you need to re-sow pastures?

- Yes (1)
- No (2)

Q7.4b Which paddocks need to be re-sown? (paddock names as listed on your paddock map)

____

____

Q7.5a Did you seek professional advice when deciding on recovery strategies?

- Yes (1)
- No (2)

Q7.5b What type of professional adviser did you seek recovery strategy advice from?

____

Q7.6 What is your estimate of the total cost of fire on your farm (in the 2019-20 fire season)?

____

Q7.7 Have you changed your fire plan since this fire? If so, how?

____

____

Q7.8 What lessons have you learnt from this fire season? What would you do differently next time?

____

Q7.9 In the next sections, we'll talk in detail about the effects on your livestock, including burns, health, reproduction and nutrition. Apart from those topics, are there any other aspects of your fire recovery that you think are important that we haven't discussed yet?

____

____

End of Block: 7 Fire recovery

Start of Block: 8 Burnt livestock

Q8.0 Please read the following text to the participant:
"That's the end of that section, we're more than halfway now. The next section of the interview is about the animal health and production effects you observed in your animals following the fire. We'll start with talking about burnt or singed animals, and then talk about other health and production effects you saw."

Check in if the participant needs a break as required.

Q8.1 Did you have any burnt livestock?

- Yes (1)
- No (2)

Q8.2 Did you have any singed livestock?

- Yes (1)
- No (2)

Q8.3 How many burnt and/or singed animal of each of the following species did you have?
(only relevant columns will appear)

	Number of animals burnt (1)	Number of animals singed (2)
Beef cattle (1)		
Dairy cattle (2)		
Sheep (3)		
Goats (4)		
Alpacas (5)		

Q8.4 Of the burnt animals, how many had each of these outcomes:

	Died in paddock due to burns on 'study day'? (1)	Died in paddock on other fire days (2)	Were destroyed (put down/shot) due to burns? (3)	Died from burns in the days/weeks after the fire (not destroyed on initial assessment) (4)	Culled due to complications or poor recovery from burns (5)	Survived burns (kept or sold for reasons not related to burns) (6)
Beef cattle (1)						
Dairy cattle (2)						
Sheep (3)						

Q8.5a Did a vet or animal health officer (or similar) visit on behalf of the government to assess your stock and/or destroy (put down/shoot) severely affected stock?

- Yes (1)
- No (2)

Q8.5b On what date(s) was/were these vet/animal health officer visit(s)?

____

Q8.6 For the burnt animals that died (not destroyed) in the days/weeks after the fire, on average how long after the fire did they die?

(If <7 days, express in days, if >7 days express in weeks, please include time units in record!)

____

Q8.7a For the burnt stock that survived, how many had each of these outcomes:

	Recovered fully and uneventfully (1)	Had complications that resolved fully (2)	Had complications that did not resolve fully (3)
Beef cattle (1)			
Dairy cattle (2)			
Sheep (3)			

Q8.7b For the animals that **recovered fully** and uneventfully, please describe the **typical pattern** of burns:

____

____

Q8.7c For the animals that had **complications that resolved** fully, please describe the **typical pattern** of burns:

____

____

Q8.7d For the animals that had **complications that resolved** fully, please describe the **complications** seen:

____

____

Q8.7e For the animals that had **complications that resolved** fully, **how long** (on average) did it take for the complications to fully resolve?

____

____

Q8.7f For the animals that had **complications that did not resolve** fully, please describe the **typical pattern** of burns:

____

____

Q8.7g For the animals that had **complications that did not resolve** fully, please describe the **complications** seen and how they progressed:

____

____

Q8.7h For the animals that had **complications that did not resolve** fully, what do you plan to do with these animals in the future?

____

____

Q8.8 Is there anything else you think is important about burns in your livestock that we haven’t discussed yet?

____

End of Block: 8 Burnt livestock

Start of Block: 9 Livestock health and production

Q9.1 (Apart from direct effects due to burns we have already discussed), Did you see any other adverse health impacts that RESULTED IN DEATHS in your stock in the 6 months after the fires?

- Yes (1)
- No (2)

Q9.2 Describe the mortality event(s). Ask additional questions to include:
What species and class of stock was affected?
How many animals died?
How many animals were affected but lived?
Were the affected animals on-farm during the fire or new stock that had been bought-in?
Briefly, what was the diagnosis (or if no diagnosis, what was observed)?
Do you think it was related to fire?

Q9.3 (Apart from effects we have already discussed including mortalities), Did you see any increase in disease or injuries that DID NOT RESULT IN DEATHS in your stock in the 6 months after the fires?

- Yes (1)
- No (2)

Q9.4 Describe the increase in disease or injuries. Ask additional questions to include:
What species and class of stock was affected?

How many animals were affected?
Were the affected animals on-farm during the fire or new stock that had been bought-in?
Briefly, what was the diagnosis (or if no diagnosis, what was observed)?
Do you think it was related to fire?

Q9.5a Have you shorn your sheep since the fire? If so, did you detect any wool-related issues that might be due to the fires?

- Yes - wool-related issues detected (e.g. tender wool) (1)
- No - no wool-related issues (2)
- I have not shorn since the fires (3)

Q9.5b Please describe the wool-related issues detected:

____

____

Q9.6a Did you change your time of shearing in 2020 due to effects of the fire?

- Yes (1)
- No (2)

Q9.6b Please describe how your time of shearing changed in 2020 due to effects of the fire:

____

____

Q9.7a Did effects of fire on infrastructure (such as yards) mean you were unable to undertake routine animal health or management procedures at the required time in 2020?
(for example, could not vaccinate, drench, shear, mark/castrate, etc.)

- Yes (1)
- No (2)

Q9.7b What routine animal health or management procedures were unable to be completed at the required time, and list any consequences of this that were observed:

____

____

Q9.8a Did effects of fire on access or infrastructure (e.g. yards) mean you were unable to treat injured or unwell animals?

- Yes (1)
- No (2)

Q9.8b Briefly describe the consequences of not being able to treat these injured or unwell animals:

____

____

Q9.9 Do you have any stock that were present during the fires still on farm at present and will be sold for slaughter in the next 6 months?

- Yes (1)
- No (2)

Q9.10 Is there anything else you think is important about the health of your livestock related to the fires that we haven't discussed yet?

____

____

End of Block: 9 Livestock health and production

Start of Block: 10 Livestock reproduction and management calendar

Q10.0 Please read the following text to the participant:
"That's the end of that section. The next section of the interview is about the effects of fire on your animals' reproduction, nutrition and biosecurity."

Check in if the participant needs a break as required.

Q10.1a Have each of the following activities have occurred during or since the fire on your farm, and if so what month(s) did they occur?

	Has this occurred?			If yes, what month(s) did it occur?
	Yes (1)	No (2)	Not relevant to my enterprise (3)	Month(s) (1)
Cattle - joining (1)				
Cattle - preg testing (2)				
Cattle - calving (3)				
Cattle - weaning (4)				

Q10.1b Have each of the following activities have occurred during or since the fire on your farm, and if so what month(s) did they occur?

	Has this occurred?			If yes, what month did it occur?
	Yes (1)	No (2)	Not relevant to my enterprise (3)	Month(s) (1)

Sheep - joining (1)				
Sheep - scanning (preg testing) (2)				
Sheep - lambing (3)				
Sheep - marking (4)				

Q10.2a Have you detected any abortions in your cattle since the fire?

- Yes (1)
- No (2)

Q10.2b Abortions in cattle: In the last 3 years prior to the fire, what % abortions in cattle have you seen on average? (also OK to provide average number of abortions / typical size of breeding herd)

____

Q10.2c Abortions in cattle: Since the fire, what number of abortions in cattle have you seen? How many breeding cows were still on-farm when these abortions were observed?

	Number of abortions seen (1)	Breeding cows on-farm at time (average) (2)
For breeding cows (1)		

Q10.3a What month(s) do you usually join your cows?

- January (1)
- February (2)
- March (3)
- April (4)
- May (5)
- June (6)
- July (7)
- August (8)
- September (9)
- October (10)
- November (11)
- December (12)

Q10.3b Did you change your cattle joining period due to the fire or its after-effects?

- Yes (1)
- No (2)

Q10.3c Why did you change your joining period?

- Unable to put bulls in at usual time (1)
- Unable to remove bulls at usual time (2)
- Cows not cycling at usual time (so allowed extra/different time for joining) (3)
- Unintended joining (e.g. fence damage failed to keep bulls out) (4)
- Other (describe below) (5)

Q10.3d What was the other reason why you changed your joining period?

____

____

Q10.3e Have the cows joined unintentionally joined been preg tested?

- Yes (1)
- No (2)

Q10.3f How many of the suspect unintentionally joined cows were found to be pregnant? What month are they expected to calve?

____

____

Q10.3g How many suspect unintentionally joined cows are there? What month are they expected to calve?

____

____

Q10.3h Over the next 3 years, will you change your joining time again to manage when your cows will calve? If so, what do you intend to do?

____

____

Q10.3i Did you join heifers?

- Yes (1)
- No (2)

Q10.3j For the heifers joined, were there more than usual that did not meet their target joining weight?

- Yes (1)
- No (2)

Q10.3k In the last 3 years compared to the time since the fire, what % of your heifers met their target joining weight?

	Since fire (1)	Last 3 years before fire (average) (2)
% not meeting target joining weight (1)		

Q10.3l Did the fire or after-effects have any other effects on cattle joining?

____

____

Q10.4 In the last 3 years compared to the time since the fire, what % of cows were preg tested in calf?

	Since fire (1)	Last 3 years before fire (average) (2)
% in calf (1)		

Q10.5 In the last 3 years compared to the time since the fire, what % of cows joined had a live calf?

	Since fire (1)	Last 3 years before fire (average) (2)
% with live calf (1)		

Q10.6a Did you wean calves at a different time since the fire to usual?

- Yes (1)
- No (2)

Q10.6b Was this change in time of weaning related to the effects of the fire on your farm?

- Yes (1)
- No (2)

Q10.7a Have you detected any abortions in your sheep since the fire?

- Yes (1)
- No (2)

Q10.7b In the last 3 years compared to the time since the fire, what % of ewes aborted?

	Since fire (1)	Last 3 years before fire (average) (2)
% ewes aborted (1)		

Q10.8a What month(s) do you usually join your ewes?

- January (1)
- February (2)
- March (3)
- April (4)
- May (5)
- June (6)
- July (7)
- August (8)
- September (9)
- October (10)
- November (11)
- December (12)

Q10.8b Did you change your sheep joining period due to the fire or its after-effects?

- Yes (1)
- No (2)

Q10.8c Were any ewes joined unintentionally following the fire? (e.g. due to damaged fencing allowing ram access)

- Yes (1)
- No (2)

Q10.8d Have the unintentionally joined ewes been scanned?

- Yes (1)
- No (2)

Q10.8e What % of the suspect unintentionally joined ewes were scanned as pregnant? What month are they expected to lamb?

____

____

Q10.8f How many suspect unintentionally joined ewes are there? What month are they expected to lamb?

____

____

Q10.8g What month(s) do you usually lamb?

- January (1)
- February (2)
- March (3)
- April (4)
- May (5)
- June (6)
- July (7)
- August (8)
- September (9)
- October (10)
- November (11)
- December (12)

Q10.8h Did the fire or after-effects have any other effects on sheep joining?

____

____

Q10.9a Do you scan your ewes single vs. multiples or just pregnant vs. empty?

- Single vs. multiples (1)
- Pregnant vs. empty (2)

Q10.9b In the last 3 years compared to the time since the fire, what were your % ewe scanning results?

	Since fire (1)	Last 3 years before fire (average) (2)
% multiples (1)		
% singles (2)		
% empty (3)		

Q10.9c In the last 3 years compared to the time since the fire, what were your % ewe scanning results?

	Since fire (1)	Last 3 years before fire (average) (2)
% pregnant (1)		
% empty (2)		

Q10.10 Were there any observations at lambing different to typical years that may have been attributable to the effects of fire? If so, please describe.

____

Q10.11 In the last 3 years compared to the time since the fire, what was your marking %?

	Since fire (1)	Last 3 years before fire (average) (2)
% lambs marked (1)		

Q10.12 Is there anything else you think is important about the reproduction of your livestock related to the fires that we haven't discussed yet?

____

____

End of Block: 10 Livestock reproduction and management calendar

---

Start of Block: 11 Livestock nutrition

Q11.3 Regarding the stock you sent for agistment, would you have sent them for agistment in 2020 anyway (e.g. routinely or due to drought)?

- Yes (1)
- No (2)

Q11.4 What date (or week/month) was the first rain since the fire that produced a green pick?

____

Q11.5 What date (or week/month) was there a break in the season? (i.e. that allowed reduction in hand feeding, or autumn break if relevant to region)

____

Q11.6 If pasture has recovered, how long did it take for burnt pasture to recover (on average across farm)?

- (1)
- 1-3 months (between one to three months after fire) (2)
- 3-6 months (between three to six months after fire) (3)
- >6 months (greater than six months after fire but recovered now) (4)
- Burnt pasture has not recovered (5)

Q11.7a Was there any storm-related damage after the fire to:

	Yes (1)	No (2)
Burnt paddock pastures or topsoil (1)		
Water sources e.g. contamination (2)		

Q11.7b For storm-related damage to pastures or topsoil, which paddocks were affected? (paddock names as listed on your paddock map)

____

____

Q11.7c For storm-related damage to water sources, which paddocks water was affected? (paddock names as listed on your paddock map)

____

____

Q11.8 Is there anything else you think is important about the nutrition of your livestock related to the fires that we haven't discussed yet?

____

____

End of Block: 11 Livestock nutrition

Start of Block: 12 Biosecurity

Q12.1 Due to fence damage in the 3 months after the 'study day', did your stock (usually kept separate) mix with each other? (e.g. groups normally kept separate allowed to run together)

- Yes (1)
- No (2)
- No fence damage occurred (3)

Q12.2a For stock that mixed, once you could separate them, did you quarantine these stock?

- Yes (1)
- No (2)

Q12.2b How long did you quarantine the stock that mixed for? (specify units as days/months)

____

Q12.2c For groups of stock that mixed, have any new diseases (not previously detected in that group) been detected since the fires?

- Yes (1)
- No (2)

Q12.3 Due to fence damage in the 3 months after the 'study day', did your stock mix with any NEIGHBOURS stock (in a way that is not usual or intended)?

- Yes (1)
- No (2)
- No fence damage occurred (3)

Q12.4a For stock that mixed with neighbours, once you could separate them, did you quarantine these stock?

- Yes (1)
- No (2)

Q12.4b For the stock that mixed with neighbours, once you could separate them, how long did you quarantine the stock? (specify units as days/months)

____

Q12.4c Have any new diseases been detected in groups of stock that mixed with neighbours' stock since the fires?

- Yes (1)
- No (2)

Q12.5a For animals purchased since the fire:

	Yes (1)	No (2)
Did you buy sheep? (1)		
Did you buy cattle? (2)		
Were they quarantined on arrival? (3)		
Did they receive any quarantine drenches? (4)		
Did you receive animal health statements for these stock? (5)		
Were there any biosecurity issues that occurred with these stock? (6)		

Q12.5b Where did you source these stock from? e.g. saleyard, Auctions Plus, private purchase, other (if other please describe)

____

Q12.5c How long were the animals quarantined for?

____

Q12.5d What quarantine drenches (product or active ingredient) were given? Include both worm and fluke drenches if relevant.

____

____

Q12.6 Were there any other consequences of the fire on farm biosecurity that that we haven't discussed yet?

____

____

Q12.7 We're at the end of the sections about the fire. Is there any other aspect of the fire on your farm that you consider important, that we haven't discussed yet?

____

____

End of Block: 12 Biosecurity

Start of Block: 13 Disease testing history

Q13.0 Please read the following text to the participant: (IF NO CATTLE SAMPLING due to all stock lost - just select "no cattle sampling" on next question and skip this section)
"That's the end of that section. There are just two sections left to go. The next section is about your animals history of specific diseases linked to the samples we're taking from your cattle today."

Check in if the participant needs a break as required.

Q13.0 Are the cattle samples collected today being tested for the following conditions:

- Pestivirus (BVD) (1)
- Faecal (worm) egg counts (2)
- No cattle sampling occurring (3)

Q13.1a Have the cattle we're sampling ever been vaccinated with Pestiguard previously? (this is the vaccine against Pestivirus/BVD)

- Yes (1)
- No (2)
- Unsure (3)

Q13.1b What month and year was the most recent Pestiguard vaccine for these animals?

____

Q13.1c Have stock on your farm ever been tested for pestivirus (also known as BVD)?

- Yes (1)
- No (2)
- Unsure (3)

Q13.1d What month and year was this testing most recently done, and were there any positive animals in those results?

____

Q13.1e To your knowledge, has a pestivirus test ever come back positive for stock on your farm? (this includes either antigen or antibody tests)

- Yes (1)
- No (2)
- Unsure (3)

Q13.1f Which of these statements best describes your stock? Prior to the fires, I'm:

- very certain they had *never* been exposed to pestivirus before (1)
- moderately certain they had *never* been exposed to pestivirus before, but I couldn't be sure (2)
- not sure at all whether they had been exposed to pestivirus (3)
- moderately certain they *had* been exposed to pestivirus before, but I couldn't be sure (4)
- very certain they *had* been exposed to pestivirus before (5)

Q13.2a On what date did the cattle we're sampling last receive a drench?

(If > 6 weeks ago and exact date not known, specify month and year)

____

Q13.2b What was the name of the drench given? (brand name or active ingredients if known)

____

Q13.2c Are you aware of any drench resistance present that affects cattle on your farm?

- Yes (1)
- No (2)

Q13.2d Please give details of drench resistance present on your farm.

____

____

End of Block: 13 Disease testing history

Start of Block: 14 Respondent demographic and contact details

Q14.0 Please read the following text to the participant:
"This is the last section - we just need to confirm some of your details and contact information, and then we'll be finished with the interview."

Q14.1 What is your age?

____

Q14.2 What is your gender
- Male (1)
- Female (2)
- Other (3)
- Prefer not to have recorded 4)

Q14.3
We are looking for fire-affected animals that will be slaughtered and processed in the remaining months of 2020. Would you be interested in allowing researchers to follow the cattle you intend to sell through to processing? If you agree, you will be provided with meat quality and animal health feedback from those animals. If you agree, Dr Melanie Smith from the University of Sydney will contact you in the next few weeks, using the contact details you've already given us.
- Yes (1)
- No (2)

Q14.4 A small number of case studies are being included in the manual that will be produced at the end of this project. Would you be interested in telling the story of your farm and recovery as a case study? If you're selected as a case study, we'll use the contact details you've already given us to contact you again about this in a few months.
- Yes (1)
- No (2)

Q14.5 Would you like us to email you a summary of the project findings when they are made available? If you agree, we'll use the email address you've already provided.
- Yes (1)
- No (2)

Q14.6 For interviewer: That's the end of the interview. Any additional information or comments to the research team about this interview can be recorded in the box below.

Please thank the farmer for their input and patience to complete this interview.

PLEASE ENSURE the participant has a hard-copy of the University of Melbourne letterhead Plain Language Statement, and take a moment now to point out the contact details for mental health support services that are listed. Please ensure the participant appears to be safe before you leave the farm today.
