# Supplementary material for: Livestock Farm Recovery Following Bushfire in South-Eastern Australia: Impacts on Cattle and Sheep Health and Management
Source: Animals (Basel). 2025 Jun 14;15(12):1764. doi: 10.3390/ani15121764 (PMC12189280; doi:10.3390/ani15121764)
Supplement: Supplementary file 1 [file animals-15-01764-s001.zip › S2_Bushfire_onlinesurvey.pdf]

Livestock and Australian Bushfires. Affected Farmer Online Survey

Health, welfare and biosecurity of livestock exposed to Australian bushfires: Online survey of affected producers.

This survey is for **Australian farmers producing beef cattle and/or sheep whose farm burnt in the 2019-20 bushfire season**. Responses from farms in all fire-affected states of Australia are welcome. If you are not a fire-affected beef or sheep farmer in Australia, thank you for your interest but please do not complete the survey. You can visit https://www.mla.com.au/bushfire-livestock-wellbeing for general information about this research project. If you participated in a research interview about your livestock health after the bushfire with NSW Local Land Services, Ausvet Pty Ltd, The University of Melbourne or The Mackinnon Project between September 2020 and January 2021, you have already answered many of these questions and do not need to complete this survey.

The survey contains questions about the effects of the 2019-20 bushfires on your farm. The survey is **confidential,** and your identity will be **anonymous** in all results reported, although the numbers of participating farms in each region of Australia will be reported. This MLA funded study is being conducted by Dr Caitlin Pfeiffer, Dr Megan Thomas, Dr John Webb Ware and Dr Carolina Munoz, at the University of Melbourne Faculty of Veterinary and Agricultural Sciences; Dr Brendan Cowled at Ausvet Pty Ltd; and Dr Karren Plain and Dr Melanie Smith at the University of Sydney. All data will be kept securely in the Faculty of Veterinary and Agricultural Sciences at the University of Melbourne for five years from the date of publication before being destroyed.

Each affected farm should only complete the survey once, even if you had multiple blocks of land affected. The survey should be completed by the person with the greatest knowledge of the consequences of the fire on the animals and farm business, in most cases this will be the **farm manager or owner-manager**. It's ok for multiple people to contribute to answering the questions, but please only complete one survey per farm. The survey should be completed in one sitting and will take 20 to 45 minutes to complete. **Participation in this study is completely voluntary and you are free to withdraw at any stage of the survey.**

For more information about the study and your privacy, please read the Plain Language Statement here.

The survey asks for some specific details about your farm. You may find it useful to have any **records of these details on-hand** as you complete the survey, so **before you start the survey,** please take a moment to review these questions and retrieve any records
This project has been approved by the University of Melbourne Ethics Committee. Should you have any concerns about the ethical conduct of the project, you are welcome to contact the Executive Officer, Human Research Ethics, The University of Melbourne, on telephone: 03 8344 2073 or email: HumanEthics-complaints@unimelb.edu.au

Q0.1
Do you consent to participate in this survey? By clicking "Yes" you acknowledge that you have read and understood the above information.

- Yes (1)
- No, thank you (2)

Q0.2 What is your relationship to the property you will be reporting about?

- Owner (1)
- Manager (2)
- Share-farmer (3)
- Other (please specify) (4)

Q0.3 **Prior to the 2019-20 fire season** on your farm located in Australia, did your stock numbers meet the following criteria:

- 10 or more beef cattle (1)
- 200 or more sheep (2)
- None of the above (3)

Q0.4 In the 2019-20 fire season, was there bushfire **on your farm land**?
Bushfire is defined as an uncontrolled fire that occurs in forest, scrub, woodland, grassland or pasture.

- Yes (6)
- No (7)

Q0.5
Thank you. Your farm meets the criteria to participate this study.
The survey will commence on the next screen.

Instructions
Please answer all questions to the best of your ability. If you do not know the answer to a question, please give us your best estimate or leave it blank. There are no "right" or "wrong" answers to any of these questions, just answer what is true for you. **Your individual responses will remain strictly confidential. Only summary results for the entire survey will be reported.**

End of Block: 0 Pre-survey checks

Start of Block: 1 Farm location, fire occurrence, fire area and intensity, and fire response

Q1.0 *The first question asks for the roadside address of your farm. This will be used to match your location to environmental data such as the nearest weather station records, drought index, etc. Your address will be kept confidential. Location will only report in the results of this study by*

locality and state. Your address will not be used to contact you unless you give specific permission at the end of the survey. Please ensure your address is complete and accurate.

Q1.1
What is the roadside address of the property you will be reporting about?

Please include number and road name, locality/suburb, state and postcode. If multiple blocks were affected by fire, list the block that was worst affected and answer the remaining questions in the survey just for that block.

____

____

The following questions relate to details of the fire. If multiple blocks were affected by fire, please answer just for the worst affected block on your farm.

Q1.2
For how many days did bushfire burn on your farm in the 2019-20 bushfire season?

____

JS

Q1.4
What date was your 'worst fire day' in the 2019-20 fire season?
This is the day the fire did the greatest damage or posed greatest threat to your property. If you only had one day of fire, enter that date.
Click to open calendar and select a date:

____

JS

Q1.5 In what hour on that 'worst fire day' did the fire start burning (or flare up your property if it was already burning)?
Click to select a time:

____

Q1.7 What were the main reason(s) fire ignited on (or nearest) your farm on the 'worst fire day'?
You may select one or more reasons.

- Main fire front reached farm (1)
- Spot fires (2)
- Persistent embers (3)
- Backburning (4)
- Lightning strike (5)
- An existing fire flared up (6)
- Other (please specify) (7)

The following questions relate to intensity of the fire:

Q1.8 What proportion (percentage) of the total grazing area of the farm was burnt?

____

Q1.9 On the 'worst fire day', did areas of pasture burn?

- Yes (1)
- No (2)

Q1.10 What is your estimate of how fast the MAIN fire moved on pasture?

- Fast (>5 km/hr i.e. faster than walking pace) (1)
- Medium (>1 to (2)
- Slow ((3)

Q1.11 What is your estimate of the height of the flame length on pasture (in metres, to nearest 0.5m)?

____

Q1.12 On the 'worst fire day', did wooded areas of the farm burn?

- Yes (1)
- No (2)

Q1.13 What is your estimate of how fast the MAIN fire moved in wooded areas?

- Fast (>5 km/hr i.e. faster than walking pace) (1)
- Medium (>1 to (2)
- Slow ((3)

Q1.14 What is your estimate of the height of the flame length in wooded areas (in metres, to nearest 0.5m)?

____

Q1.15 What is your estimate of the average depth of litter (fine fuel) on the ground in wooded areas (in cm)?

To assist your estimation, for example, the height of the rubber on your boot's sole may be approx 2cm, ankle height may be approx 12cm, knee height may be approx 55cm.

____

Q1.16 How wide was the MAIN fire front?

- Spot fires or narrower than a paddock (i.e. (1)
- Broad front at least a paddock wide (i.e. >400m) (2)

Q1.19 Did you have a fire plan in place at the time of the fires?

- Yes - written fire plan (3)
- Yes - thought or verbal only, not written (4)
- No (5)

Q1.20 In the days before the fire did you undertake specific activities to prepare for the fire?

- Yes (please list any specific preparation activities here) (1) ____
- No (2)

Q1.21 Were active efforts to fight the fire made when your property was in fire?

This includes firefighting by you or others on the farm, as well as the local fire authority if they were able to attend and help fight the fire (CFA, RFS, CFS, QFES, etc.).

- Yes (1)
- No (2)

End of Block: 1 Farm location, fire occurrence, fire area and intensity, and fire response

---

Start of Block: 2 Farm type and management

Q2.0
The following questions relate to details of your farm.
The first questions in this section relate to the number, type and characteristics of the stock on your farm on your 'worst fire day' in the 2019-20 summer. If you have **multiple blocks, just answer this section for the block worst affected by fire.**

Q2.1 Which types of production animals did you have on the block?

- Beef cattle (1)
- Dairy cattle (2)
- Sheep (3)
- Goats (4)
- Alpacas (5)

Q2.2a What type of dairy enterprise did you have?

If you are unsure, hover over or click underlined text for definition of each type.

- Seasonal calving (1)
- Split calving (2)
- Year-round calving (3)

Q2.2b

What type of beef enterprise did you have?

You can select multiple types. If you are unsure, hover over or click underlined text for definition of each type.

- Self-replacing commercial (1)
- Self-replacing seedstock (2)
- Growing and backgrounding (4)
- Finishing on pasture (7)
- Finishing in feedlot (8)
- Trading (3)
- Agistment (5)
- Other (please specify) (6)

Q2.2c

What type of sheep enterprise did you have?

You may select multiple types.

- Self-replacing merino (1)
- Self-replacing prime lamb (2)
- Terminal sire to merino (breeding first cross ewes or terminal lambs) (3)
- Terminal sire to first cross ewes (4)
- Stud (5)
- All wethers (6)
- Trading e.g. trade lambs (7)
- Other (please specify) (8)

Q2.3a
How many cattle did you have in each of the following groups on the 'worst fire day' on this block? *Please enter response in digits, e.g. 1,2,3...*

	Number of animals (1)
Unweaned calves (1)	
Weaned calves including vealers (up to 12 months) (2)	
Yearlings & heifers (12 months to 2 years) (3)	
Prime cattle/bullocks (2-4 years) (4)	
Cows (>2 years) (5)	
Bulls (>2 years) (6)	

Q2.3b
How many sheep did you have in each of the following groups on the 'worst fire day' on this block? *Please enter response in digits, e.g. 1,2,3....*

	Number of animals (1)
Unweaned lambs (1)	
Weaners (up to 12 months) (2)	
Hoggets & ewe lambs (12 months to 2 years) (3)	
Adult ewes (>2 years) (4)	
Adult rams (>2 years) (5)	
Adult wethers (>2 years) (6)	

Q2.3c
How many of these other species did you have in each of the following groups on the 'worst fire day' on this block? *Please enter response in digits, e.g. 1,2,3....*

	Number of animals (1)
Alpacas (total) (1)	
Dairy goats (total) (2)	
Non-dairy goats (total) (3)	

Q2.4 The next question is about the area of your farm (or block that was worse fire-affected). What unit will you report farm size in?

- Hectares (1)
- Acres (2)

Q2.5
What is the area of the block (in ${Q2.4/ChoiceGroup/SelectedChoices}) for each of the following purposes? *Please enter response in digits, e.g. 1,2,3...*

	Land area (${Q2.4/ChoiceGroup/SelectedChoices}) (1)
Total area (1)	
Area grazing land (includes fodder crops) (2)	
Area with commercial crops (3)	
Area bush block/other (4)	

Q2.6 How did you graze your stock in spring and summer 2019, prior to the fire?

- Set stocking (1)
- Rotational grazing (2)
- Both set stocking and rotational grazing (3)

Q2.7 Given the stocking rate on your farm and the landscape of your farm, was your stocking rate before the fire:

- High (1)
- Medium (2)
- Conservative (3)

Q2.8 Do you irrigate pasture on this block or farm?

- Yes (1)
- No (2)

Q2.10 Which of the following best describes your farm in the first month after the 'worst fire day'?

- All stock lost or removed from property after fire (1)
- Some stock lost or removed from property after fire (2)
- No stock lost or removed from property after fire (3)

Q2.11
When considering the stock that were lost or removed from the property, were they:
You may select multiple answers if appropriate

- Sent for agistment on another property owned by you (2)
- Sent for agistment on another property owned by someone else (3)
- Sold through saleyards (4)
- Sold to abattoirs (5)
- Killed in the fire or put down (eg shot) due to burns or other injury/disease (1)

Q2.14 Have you purchased new stock since the fire?

- Yes (1)
- No (2)

Q2.15 Which of the following best describes your farm now?

- No stock remaining on-farm (1)
- Reduced stock numbers compared to the last 3 years (2)
- Similar stock numbers compared to the last 3 years (3)
- Increased stock numbers compared to the last 3 years (4)

Q2.17 Have you joined **cows** since the fires?

- Yes, at the usual time (25)
- Yes, at a different time to usual (26)
- No (27)

Q2.18
Why did you change your **cattle joining** period?
You may select multiple options.

- Unable to put bulls in at usual time (1)
- Unable to remove bulls at usual time (2)
- Cows not cycling at usual time so allowed additional time for joining (3)
- Unintended joining due to infrastructure damage (e.g. fences lost) (4)
- Other (please describe briefly) (5)

Q2.19a Did you change the time of **calf weaning** in 2020?

- Yes - earlier than usual (1)
- Yes - later than usual (2)
- No change (3)
- Have not weaned in 2020 (4)

Q2.19b Did you change the time of **lamb weaning** in 2020?

- Yes - earlier than usual (1)
- Yes - later than usual (2)
- No change (3)
- Have not weaned in 2020 (4)

Q2.20 Were your stock receiving supplementary feed in spring and summer 2019, prior to the 'worst fire day'?

- Yes (23)
- No (24)

Q2.21 Did your stock receive supplementary feed in the 6 months after the fire?

- Yes (49)
- No (50)

Q2.23
What type(s) of supplementary feed did you provide?
Please select all types provided.

- Straw (3)
- Hay (1)
- Silage (17)
- Grain - wheat (19)
- Grain - barley (20)
- Grain - oats (21)
- Grain - lupins (22)
- Grain - other (please describe) (27)
- Pellets (23)
- Other (please describe) (24)

Q2.24 What month was the first rain since the fire that produced a green pick?

▼ January (4) ... December (15)

Q2.25 What month was there a break in the season after the fire? *(i.e. rain that allowed reduction in hand feeding, or autumn break if relevant to region)*

▼ January (4) ... December (15)

Q2.26 If pasture has recovered, how long did it take for burnt pasture to recover after it was burnt (on average across farm)?

- (1)
- 1-3 months (between one to three months after fire) (2)
- 3-6 months (between three to six months after fire) (3)
- >6 months (greater than six months after fire but recovered now) (4)
- Burnt pasture has not recovered (5)

Q2.27
For pastures that have regrown since the fire, has the composition of the burnt pastures changed compared to the last 3 years?
For example, a change in the proportion of clover compared to grasses or change in dominant grass species.

- No (1)
- Yes (please describe) (2)

  ____

- Not sure (3)

Q2.28 Was there any storm-related or other damage after the fire to:

	Yes (1)	No (2)
Burnt paddock pastures or topsoil (1)		
Water sources e.g. contamination (2)		

End of Block: 2 Farm type and management

Start of Block: 3 Burnt livestock

Q3.0 The following questions relate to details of animals that were burnt or singed during the fire.

Q3.1 Did you have any burnt livestock?

- Yes (1)
- No (2)

Q3.2 Did you have any singed (but not burnt) livestock?

- Yes (1)
- No (2)

Q3.3 How many burnt and/or singed animal of each of the following species did you have? (only relevant columns will appear)
Please enter response in digits, e.g. 1,2,3...

	Number of animals burnt (1)	Number of animals singed only (2)
Beef cattle (1)		
Dairy cattle (2)		
Sheep (3)		
Goats (4)		
Alpacas (5)		

Q3.4 When considering the **burnt cattle,** which parts of the body were affected?

- Face and/or horns (1)
- Feet +/- legs (2)
- Teats +/- udder (3)
- Breech/hind end (4)
- Burnt all over (6)
- Other (please specify below) (5)

____

Q3.6a Of the **burnt cattle**, how many had each of these outcomes:
You indicated previously you had a total of ${Q3.3/ChoiceTextEntryValue/1/1} beef cattle burnt and ${Q3.3/ChoiceTextEntryValue/2/1} dairy cattle burnt.

	Died in paddock due to burns on day of fire? (1)	Were destroyed (put down/shot) due to burns? (3)	Died from burns in the days/weeks after the fire (not destroyed on initial assessment) (4)	Culled due to complications or poor recovery from burns (5)	Survived (kept or sold for reasons not related to burns) (6)
Number of cattle (1)					

Q3.6b Of the **burnt cattle**, how many had each of these outcomes:
You indicated previously you had a total of ${Q3.3/ChoiceTextEntryValue/1/1} beef cattle burnt.

	Died in paddock due to burns on day of fire? (1)	Were destroyed (put down/shot) due to burns? (3)	Died from burns in the days/weeks after the fire (not destroyed on initial assessment) (4)	Culled due to complications or poor recovery from burns (5)	Survived (kept or sold for reasons not related to burns) (6)
Number of cattle (1)					

Q3.6c Of the **burnt cattle**, how many had each of these outcomes:
You indicated previously you had a total of ${Q3.3/ChoiceTextEntryValue/2/1} dairy cattle burnt.

	Died in paddock due to burns on day of fire? (1)	Were destroyed (put down/shot) due to burns? (3)	Died from burns in the days/weeks after the fire (not destroyed on initial assessment) (4)	Culled due to complications or poor recovery from burns (5)	Survived (kept or sold for reasons not related to burns) (6)
Number of cattle (4)					

Q3.5 When considering the **burnt sheep**, which parts of the body were affected?

- Face and/or horns (1)
- Feet +/- legs (2)
- Teats +/- udder (3)
- Breech/hind end (4)
- Burnt all over (6)
- Other (please specify below) (5)

____

Q3.7 Of the **burnt sheep**, how many had each of these outcomes:
You indicated previously you had a total of ${Q3.3/ChoiceTextEntryValue/3/1} sheep burnt.

	Died in paddock due to burns on day of fire? (1)	Were destroyed (put down/shot) due to burns? (3)	Died from burns in the days/weeks after the fire (not destroyed on initial assessment) (4)	Culled due to complications or poor recovery from burns (5)	Survived (kept or sold for reasons not related to burns) (6)
Number of sheep (1)					

Q3.8 Did you observe any flystrike in burnt stock? If so, which species was affected?

- No flystrike observed in any species (6)
- Sheep affected (7)
- Cattle affected (8)
- Goats affected (10)
- Alpacas affected (11)

End of Block: 3 Burnt livestock

Start of Block: 4 Livestock health and production

Q4.0 The following questions relate to details of your animals' health and production in the 12 months after the fire.

Q4.1 Apart from direct effects due to burns, did you see any other diseases or health problems that resulted in **increased deaths** in your stock in the 12 months after the fires?

- Yes (1)
- No (2)

Q4.3
Apart from direct effects due to burns, did you see any **increases in diseases or injuries that did not cause death** in your stock in the 12 months after the fires?

- Yes (1)
- No (2)

Q4.5a

In the **cattle** that were on the property during the fire, did you notice any change in rates of the following conditions, in the first 12 months after the fire (compared to 2017-2019)?

Select *the option on the scale that most closely matches your observation. If you didn't see any of that condition since the fire, think about whether this is the same as 2017-2019 (select no change) or if it is a decrease compared to 2017-2019 (select somewhat or much decreased).*

	Much decreased compared to 2017-2019 (1)	Somewhat decreased compared to 2017-2019 (2)	No change compared to 2017-2019 (3)	Somewhat increased compared to 2017-2019 (4)	Much increased compared to 2017-2019 (5)	Don't know (6)
Acidosis (grain overload) (1)						
Respiratory disease (such as coughing or pneumonia) (2)						
Eye diseases (such as pinkeye or third eyelid irritations) (3)						
Abnormalities in calves born since the fire (such as birth defects, more disease or lower growth) (4)						
Plant toxicities or poisonings (including both pasture and weeds) (16)						
Lameness or sore feet (other than healing burns in first 6 weeks) (19)						
Click to write Statement 9 (20)						
Unexplained calf deaths (17)						
Unexplained deaths in cattle other than calves (18)						

Q4.5b
In the **cows** that were on the property during the fire, did you notice any change in the following reproductive measures, in the first 12 months after the fire (compared to 2017-2019)?

***Select** the option on the scale that most closely matches your observation. If you didn't see any of that condition since the fire, think about whether this is the same as 2017-2019 (select no change) or if it is a decrease compared to 2017-2019 (select somewhat or much decreased).*

	Much decreased compared to 2017-2019 (1)	Somewhat decreased compared to 2017-2019 (2)	No change compared to 2017-2019 (3)	Somewhat increased compared to 2017-2019 (4)	Much increased compared to 2017-2019 (5)	Don't know / Not measured (6)
Failure to conceive (such as lower % pregnancy tested in-calf) (5)						
Calving to conception interval (15)						
Abortions (calf loss before calving due date) (6)						
Calves born dead (stillbirths and calf death during calving) (14)						

Q4.6a

In the **sheep** that were on the property during the fire, did you notice any changes in rates of the following conditions, in the first 12 months after the fire (compared to 2017-2019)?

Select *the option on the scale that most closely matches your observation. If you didn't see any of that condition since the fire, think about whether this is the same as 2017-2019 (select no change) or if it is a decrease compared to 2017-2019 (select somewhat or much decreased).*

	Much decreased compared to 2017-2019 (1)	Somewhat decreased compared to 2017-2019 (2)	No change compared to 2017-2019 (3)	Somewhat increased compared to 2017-2019 (4)	Much increased compared to 2017-2019 (5)	Don't know (6)
Acidosis (grain overload) (1)						
Respiratory disease (such as coughing or pneumonia) (2)						
Eye diseases (such as pinkeye or third eyelid irritations) (3)						
Abnormalities in lambs born since the fire (such as birth defects, more disease or lower growth) (4)						
Plant toxicities or poisonings (including both pasture and weeds) (16)						
Lameness or sore feet (other than healing burns in first 6 weeks) (17)						
Mastitis (19)						
Wool-related effects (such as tender wool) (5)						
Unexplained deaths in sheep (other than newborn lambs) (18)						

Q4.6b
In the **ewes** that were on the property during the fire, did you notice any changes in the following reproductive measures, in the first 12 months after the fire (compared to 2017-2019)?

Select *the option on the scale that most closely matches your observation.*

	Much decreased (more than 5% change) (1)	Somewhat decreased (5% change or less) (2)	No change (3)	Somewhat increased (5% change or less) (4)	Much increased (more than 5% change) (5)	Don't know / Not measured (6)
Scanning percentage (15)						
Abortions (lamb loss before lambing due date) (6)						
Lambs born dead and newborn lamb losses in days after lambing (16)						
Marking percentage (14)						

Q4.6c You reported observing increased plant toxicities or poisonings. What plant species do you think caused this? You can list multiple if relevant, and leave blank if you don't know.

____

Q4.2 Did you observe any other diseases or animal health problems **that you consider are a result of the fire** that were not included in the previous questions? If so, please briefly describe, including the type of disease or problem and its cause (if known).

You can include diseases or health problems due to direct effects of the fire, and also indirect effects of farm management during fire recovery.

____

____

Q4.4 Is there anything else that you think is important about the health of your livestock since the fire that you would like to share?

____

____

Q4.7 Thinking about your **sheep**,
were there any effects of the fire on your time of shearing in 2020?

- Yes (please describe the change) (1)
- ____
- No (2)

End of Block: 4 Livestock health and production

Start of Block: 5 Livestock welfare

Q5.0 The following questions relate to your perceptions and opinions about fire situations, including animal welfare effects.

Q5.1
Do you currently have any welfare concerns in relation to the stock the survived the fires?
You can add any further comments you have on the welfare of your stock in the box below your answer.

- Yes (please describe below) (1)
- ____
- No (2)

Q5.2 For each statement below, **select** the option on the scale that most closely represents you level of **agreement** or **disagreement** with the statement. There are no right or wrong answers, please just give your honest response.

	Strongly disagree (2)	Disagree (3)	Neither agree or disagree (4)	Agree (6)	Strongly agree (11)
The **overall productivity** of the animals that survived the fire was affected in the **short-term** (first month after fire) (18)					
The **overall productivity** of the animals that survived the fire was affected in the **long-term** (more than a month after fire) (28)					
The **overall health condition** of the animals that survived the fire was affected in the **short-term** (first month after fire) (19)					
The **overall health condition** of the animals that survived the fire was affected in the **long-term** (more than a month after fire) (20)					
The **behaviour** of the animals that survived the fire was affected in the **short-term** (first month after fire) (21)					
The **behaviour** of the animals that survived the fire was affected in the **long-term** (more than a month after fire) (22)					
Animals that survived the fires were more likely to get sick later					

(23)					
It is difficult for me to implement preventative measures for bushfires (24)					
I have experience with bushfires and I feel prepared (25)					
It is difficult for me to care for burnt stock (26)					
You can't always be prepared for bushfires (27)					

Q5.3 You indicated that the behaviour of your livestock was affected after the fires. What change(s) in animal behaviour were observed?

____

____

Q5.4 For each statement below, please **select** the option on the scale that most closely represents you. There are no right or wrong answers.

	Very difficult (1)	Difficult (10)	Neutral (11)	Easy (12)	Very easy (13)	Not relevant to me (14)
How difficult is it for you to be prepared for bushfires? (25)						
How difficult is it for you to get access to firefighter support? (26)						
How difficult is it for you to move stock in advance of a fire? (27)						
How difficult is it for you to have a fire plan for your property? (28)						
How difficult is it for you to determine which animals will be evacuated in an emergency? (29)						
How difficult is it for you to move stock in response to a fire? (30)						
How difficult is it for you to care for burnt stock? (31)						
How difficult is it for you to have a fire recovery plan? (32)						

End of Block: 5 Livestock welfare

Start of Block: 8 Biosecurity

Q172 The following questions relate to biosecurity on your farm.

Q8.0 Would you like to answer some questions about biosecurity (animal diseases and weeds) on your farm following the fire?
This section takes approximately 5 minutes to complete. Your experience after these fires is valuable and we appreciate your contribution.

- Yes (1)
- No (2)

Q8.1a Did you move stock off-farm in the 14 days before or on the 'worst fire day' for fire preparation?

- Yes (1)
- No (3)

Q8.1b Did you move stock to a safe area on-farm in the **14 days before or on** the 'worst fire day' for fire preparation?

- Yes (1)
- No (2)

Q8.4 Due to fence damage, did your stock mix with any **neighbours'** stock (in a way that is not usual or intended) in the 3 months after the 'worst fire day'?

- Yes (1)
- No (2)
- No fence damage occurred (3)

Q8.5a For stock that mixed with neighbours, once you could separate them, did you quarantine these stock (run them separately from other livestock groups)?

- Yes (please state how long for and specify units as days/weeks) (1)
- ____
- No (2)

Q8.5b You indicated that stock that mixed with neighbours's stock were not quarantined. Which of the below best describes the reason why?
If you want to make any further comments or clarification, you can enter this in the box below your selected choice.

- Considered quarantine but separating stock was not possible (e.g. due to damage to infrastructure such as fences). (1)

  ____

- Considered quarantine but decided it was not necessary for these animals. (2)

  ____

- Did not consider quarantine. (4)

  ____

- Other (please describe) (3)

  ____

Q8.5c Have any new diseases been detected in groups of stock that mixed with neighbours' stock since the fires?

- Yes (please name the disease(s) below) (1)
- ____
- No (2)

Q8.6a You previously indicated that stock were sent to agistment after the fire. Did these animals return to the main property after agistment? If so, did you quarantine (run them separately from other livestock groups) these stock on return and how long for?

- Stock did not return to main property (1)
- Stock returned to main property and were NOT quarantined (2)
- Stock returned to main property and were quarantined (please state how long for and specify units in days/weeks (3)

  ____

Q8.6b You indicated that stock that returned from agistment were not quarantined. Which of the below best describes the reason why?
If you want to make any further comments or clarification, you can enter this in the box below your selected choice.

- Considered quarantine but separating stock was not possible (e.g. due to damage to infrastructure such as fences). (1) ____
- Considered quarantine but decided it was not necessary for these animals. (2) ____
- Did not consider quarantine. (4) ____
- Other (please describe) (3) ____

Q8.7 You previously indicated that you have purchased new stock since the fire. What stock did you purchase?

	Yes (1)	No (2)
Did you buy sheep? (1)		
Did you buy cattle? (2)		

Q8.7a Regarding the cattle that were purchased, where did you source the cattle from?
You can select as multiple options.

- Saleyard (1)
- *AuctionsPlus* or similar online sale (2)
- Private purchase (3)
- Other (please describe below) (4) ____

Q8.7c Regarding the **cattle** that were purchased,
please answer the following questions:

	Additional	Answer	
	Comments (1)	Yes (1)	No (2)
Were these cattle quarantined on arrival, if so how long for? (5)			
Did they receive any quarantine drenches? If so, what product(s) were used. Include both worm and fluke drenches if relevant. (6)			
Did you receive animal health statements for these cattle? (7)			
Were there any biosecurity issues that occurred with these cattle? (8)			

Q8.7b Regarding the **sheep** that were purchased, where did you source the sheep from?
You can select as multiple options.

- Saleyard (1)
- *AuctionsPlus* or similar online sale (2)
- Private purchase (3)
- Other (please describe below) (4)

____

Q8.7d Regarding the **sheep that were purchased**,
please answer the following questions:

	Additional	Answer	
	Comments (1)	Yes (1)	No (2)
Were these sheep quarantined on arrival, if so how long for? (5)			
Did they receive any quarantine drenches? If so, what product(s) were used. Include both worm and fluke drenches if relevant. (6)			
Did you receive animal health statements for these sheep? (7)			
Were there any biosecurity issues that occurred with these sheep? (8)			

Q8.8
Did you notice any **new** weeds on the property after the fires?
Please include only weeds that had not previously been identified on the farm.

- Yes (please name the weeds below, if known) (1)
- ____
- No (5)

Q8.9
Did you notice any increase in the amount of **existing** weeds (those that had been seen previously on-farm) after the fire?

- Yes (please name the weeds below, if known) (1)
- ____
- No (5)

End of Block: 8 Biosecurity

Start of Block: 6A Meat and carcass quality - cattle

Q6.0 The following questions relate to details of cattle that have been slaughtered since the fire.

Q6.1 Have you sold cattle for slaughter since the fire?

- Yes (23)
- No (24)

Q6.2 Did you sell cattle for slaughter (including salvage) in the 6 weeks following the 'worst fire day'?

- Yes (23)
- No (24)

Q6.0a
Would you like to answer some questions about your cattle that were sold for slaughter in the first 6 weeks after the fire?
We appreciate your contribution.

- Yes (1)
- No (3)

Q6.3
How many cattle were sold in those first 6 weeks in each stock class?
Please enter the number of cattle sold in each category in digits, e.g. 1,2,3....

	Number of animals (1)
Vealers (1)	
Beef (2)	
Bull (3)	

Q6.4 Were the cattle sold in the first 6 weeks sold:

- Direct to abattoir for salvage slaughter (1)
- Slaughter - to abattoir direct consignment MSA (2)
- Slaughter - to abattoir direct consignment non-MSA (but not as salvage slaughter) (3)
- Saleyards - store (4)
- Saleyards - finished (5)
- Other (please describe) (6) ____

Q6.5
Did you despatch cattle **for slaughter** before they were finished?
Include only the stock that went to slaughter (not those sold on to other farms for finishing).

- No - all stock were finished (1)
- Yes - less than half the stock despatched were not finished (2)
- Yes - more than half the stock despatched were not finished (3)
- Yes - all the stock despatched were not finished (4)

Q6.6 Was the abattoir you sold cattle to in the first six weeks after the fire:

- The same abattoir you usually use (1)
- A different abattoir to usual (2)
- I use multiple abattoirs or change frequently (3)

Q6.7 What was the name and location of the abattoir(s) you sold cattle to in the first six weeks after the fire?

____

Q6.8 Did you notice or receive feedback about any meat quality issues (e.g. high pH or dark colour) in your cattle sold in the 6 weeks following the 'worst fire day'?

- Yes (please describe the issues and whether any penalties were applied) (1) ____
- No issues reported (2)
- No feedback received (3)

Q6.9 Did you notice or receive feedback about any carcass quality issues (e.g. fat depth or carcass weight) in your cattle sold in the 6 weeks following the 'worst fire day'?

- Yes (please describe the issues and whether any penalties were applied) (1) ____
- No issues reported (2)
- No feedback received (3)

Q6.10 Did you notice or receive feedback about any condemnation issues (e.g. pleurisy, pneumonia, bruising) in your cattle sold in the 6 weeks following the 'worst fire day'?

- Yes (please describe the issues and whether any penalties were applied) (1) ____
- No issues reported (2)
- No feedback received (3)

Q6.11 The next question is about the price you received for the cattle sold in the 6 weeks following 'worst fire day'. What units will you report the price in?

- per head (1)
- per kg live (2)
- per kg carcass weight (cwt) (3)

Q6.12a Using the units you just selected, what price did you receive for the vealers that you sold?

____

Q6.12b Using the units you just selected, what price did you receive for the beef cattle that you sold?

____

Q6.12c Using the units you just selected, what price did you receive for the bull animals that you sold?

____

Q6.13 How did the price you received compare to the prevailing market prices for cattle at the time?

- Discount (1)
- Equal (2)
- Premium (3)

Q6.14 In a typical year, how do you usually sell cattle for slaughter?

- To abattoir, direct consignment MSA (1)
- To abattoir, direct consignment non-MSA (2)
- Via saleyards (3)
- Other (please describe) (4)

  ____

End of Block: 6A Meat and carcass quality - cattle

Start of Block: 6B Meat and carcass quality - sheep

Q174 The following questions relate to details of sheep that have been slaughtered since the fire.

Q6.15 Have you sold sheep for slaughter since the fire?

- Yes (23)
- No (24)

Q6.16 Did you sell sheep for slaughter (including salvage) in the 6 weeks following the 'worst fire day'?

- Yes (23)
- No (24)

Q6.0b
Would you like to answer some questions about your sheep that were sold for slaughter in the first 6 weeks after the fire?
We appreciate your contribution.

- Yes (1)
- No (2)

Q6.17
How many sheep were sold in those first 6 weeks in each stock class?
Please enter the number of sheep sold in each category in digits, e.g. 1,2,3....

	Number of animals (1)
Lamb (1)	
Mutton (2)	

JS

Q6.18 Were the sheep sold in the first 6 weeks sold:
- Direct to abattoir for salvage slaughter (1)
- To abattoir direct consignment ('over the hooks' but not as salvage slaughter) (2)
- Saleyards - store (4)
- Saleyards - finished (5)
- Other (please describe) (6)

____

Q6.19
Did you despatch sheep **for slaughter** before they were finished?
Include only the stock that went to slaughter (not those sold on to other farms for finishing).
- No - all stock were finished (1)
- Yes - less than half the stock despatched were not finished (2)
- Yes - more than half the stock despatched were not finished (3)
- Yes - all the stock despatched were not finished (4)

Q6.20 Was the abattoir you sold sheep to in the first six weeks after the fire:
- The same abattoir you usually use (1)
- A different abattoir to usual (2)
- I use multiple abattoirs or change frequently (3)

Q6.21 What was the name and location of the abattoir(s) you sold sheep to in the first six weeks after the fire?

____

Q6.22 Did you notice or receive feedback about any meat quality issues (e.g. high pH or dark colour) in your sheep sold in the 6 weeks following the 'worst fire day'?
- Yes (please describe the issues and whether any penalties were applied) (1) ____
- No issues reported (2)
- No feedback received (3)

Q6.23 Did you notice or receive feedback about any carcass quality issues (e.g. fat depth or carcass weight) in your sheep sold in the 6 weeks following the 'worst fire day'?
- Yes (please describe the issues and whether any penalties were applied) (1) ____
- No issues reported (2)
- No feedback received (3)

Q6.24 Did you notice or receive feedback about any condemnation issues (e.g. pleurisy, pneumonia, bruising) in your sheep sold in the 6 weeks following the 'worst fire day'?

- Yes (please describe the issues and whether any penalties were applied) (1) ____
- No issues reported (2)
- No feedback received (3)

Q6.25 The next question is about the price you received for the sheep sold in the 6 weeks following the 'worst fire day'. What units will you report the price in?

- per head (1)
- per kg live (2)
- per kg carcass weight (cwt) (3)

Q6.26a Using the units you just selected, what price did you receive for the lambs that you sold?

____

Q6.26b Using the units you just selected, what price did you receive for the mutton that you sold?

____

Q6.27 How did the price you received compare to the prevailing market prices for sheep at the time?

- Discount (1)
- Equal (2)
- Premium (3)

Q6.28 In a typical year, how do you usually sell sheep for slaughter?
Please select as many as are relevant.

- To abattoir, direct consignment ('over the hooks') (2)
- Saleyards (3)
- Other (please describe) (4) ____

End of Block: 6B Meat and carcass quality - sheep

Start of Block: 7 Economics

Q7.0
The following questions relate to details of the costs of the fire to your farm business. This section asks for specific dollar values for a number of costs and income related to the fire. All values provided will be confidential and individual dollar values provided will not be reported except as summary statistics (e.g. average, range) and as inputs for economic models using a partial budgeting approach.

Q7.1a What is your estimate of the total cost of the fire on your farm (in the 2019-2020 fire season)?

- Less than $50,000 (4)
- $50,000 to $99,999 (5)
- $100,000 to $199,999 (6)
- $200,000 to $299,999 (7)
- $300,000 to $399,999 (8)
- $400,000 to $499,999 (9)
- $500,000 to $749,999 (10)
- $750,000 to $999,999 (11)
- $1,000,000 to $2,000,000 (12)
- $2,000,000 or more (13)
- I cannot estimate the cost (14)
- I do not wish to provide an estimate (15)

Q7.1b If you have any comments about your cost estimate, please enter them here:

____

Q7.3
Did you have insurance that would cover fire-related losses? If so, which of the following were covered?
You may select multiple options.
Select all options where a policy was held, even if no claim was needed e.g. house was insured but not lost in fire.
If a policy only covered some of that category e.g. only certain sheds insured, please still select that option.

- House (1)
- Farm infrastructure e.g. sheds (2)
- Farm equipment e.g. tractors (7)
- Livestock (3)
- Business loss (4)
- Other (please describe) (6)
  ____
- No insurance (5)

Q7.2
Are you willing to provide a breakdown of costs of the fire on your farm?
If you answer no, you will not be asked for specific dollar values and the economics section of the survey will be skipped.

- Yes (1)
- No (3)

Q7.4 Complete the below table to indicate the total dollar value of assistance received in each of the following categories due to the fire. If you don't know the exact dollar value, an estimate is OK.

	Dollar value of assistance (1)
Insurance payout (1)	
Financial grants from government or other organisations (2)	
Feed supplied by government or other organisations (3)	
Other non-financial assistance from other organisations e.g. BlazeAid (5)	

JS

Q7.5 Complete the below table to indicate the total dollar value of extra expenditure in the first 12 months **beyond what would have been otherwise expected** in each of the categories. If there was no change from usual expenditure, enter 0. If you don't know the exact dollar value, an estimate is OK.

	Additional Expenditure (AUD) (1)
Infrastructure (replacement completed) (1)	
Infrastructure (not yet completed; projected cost) (2)	
Temporary infrastructure (e.g. hired yards, short-term repairs) (3)	
Additional labour for usual farm activities (4)	
**Additional** supplementary feed (total bill for all feed types) (5)	
Consumable costs for increasing short term feed grown (e.g. oversowing, nitrogen , gibberellic acid, fodder crops), above what would have been done if no fire (6)	
Application costs for increasing short term feed grown, above what would have been done if no fire (7)	
Additional agistment costs (8)	
Freight associated with additional agistment costs (9)	

JS

Q7.6 Complete the below table to indicate the total dollar value of losses due to the fire in each of the categories. If no losses, enter 0. If you don't know the exact dollar value, an estimate is OK.

	Value of losses (AUD) (1)
Stored feed lost or no longer suitable for livestock (total value for all feed types) (1)	
Infrastructure lost (e.g. sheds, yards, fencing) (2)	
Equipment lost (3)	
Unharvested crops lost (5)	
Stubbles lost (6)	
Intensive industry on-farm lost (e.g. orchards) (4)	

Q7.8 What area (reported in ${Q2.4/ChoiceGroup/SelectedChoices}) of grazing land requires pasture renovation due to fire?

____

Q7.9
In each category, since the fire, how many additional **cattle** did you sell or buy, compared to what you would have expected if there had been no fire?
Please enter the number in digits ie 1,2,3..
PTIC = Pregnancy Tested In-Calf

	Additional number sold (1)	Additional number bought (2)
Cows with calves at foot (1)		
Weaned calves including vealers (up to 12 months) (2)		
Yearlings & heifers (12 months to 2 years) (3)		
Prime cattle/bullocks (2-4 years) (4)		
Empty cows (2 years +) with no calves at foot (5)		
PTIC cows (2 years +) with no calves at foot (7)		
Bulls (2 years +) (6)		

Q7.10
In each category, since the fire, how many additional **sheep** did you sell or buy, compared to what you would have expected if there had been no fire?
Please enter the number in digits i.e. 1,2,3…

	Additional number sold (1)	Additional number bought (2)
Weaners (up to 12 months old) (1)		
Hoggets (12 months to 2 years) (2)		
Adult ewes (2 years +) (3)		
Adult rams (2 years +) (4)		
Adult wethers (2 years +) (5)		

Q7.11
You previously indicated your livestock enterprise is currently understocked compared to the past 3 years. Which of the following best describes the reason for this?
Please select as many as are relevant to your situation.

- Changing enterprise type (1)
- Suitable animals not affordable (2)
- Suitable animals not available (3)
- Not enough feed available for typical stocking rate (4)
- Typical stocking rate not desirable due to personal circumstances or stress post-fire (5)
- Other (please describe briefly) (6)
____

Q7.12 If you have any additional comments about the responses you have provided in this section about costs and income related to the fires on your farm, please enter them here:

____

____

End of Block: 7 Economics

Start of Block: 9 Open-ended comments, demographic and contact details

Q9.0 This is the last section of the survey. The following questions ask for your final comments, your age and gender, and allow you to enter your contact details if you would like to receive a summary of the project findings.

Q9.1 Considering your livestock enterprise, if facing fire of a similar nature in the future, is there anything you would do differently? Please describe here:

____

____

Q9.2
Is there any other aspect of the fire on your farm related to beef or sheep production that you would like to comment on?

____

____

Q9.3 We are looking for farms willing to provide farm maps to investigate fire risk across farm landscapes. We would ask you to either provide existing digital maps, or we would send you paper maps to mark out. Map information will be used together with other existing data like weather records and drought index.

Would you be willing to complete a map of your farm showing details relating to stock locations at the time of the fire, burnt areas and locations of fire preparation activities like fire breaks?

If you answer yes, you will be asked for your contact details below.

- Yes (1)
- No (4)

Q9.5 Would you like us to email you a summary of the project findings when they are made available?
If you answer yes, you will be asked for your contact details below.

- Yes (1)
- No (2)

Q9.6 What is your age?
Answer this question for the main person who has provided answers for the survey. If two people equally, select the person who will be the main contact person for any future follow-up.

____

Q9.7 What is your gender?
Answer this question for the main person who has provided answers for the survey. If two people equally, select the person who will be the main contact person for any future follow-up.

- Male (1)
- Female (2)
- Other (3)
- Prefer not to have recorded (4)

Q9.8 If you agreed to be contacted in the preceding questions, please enter your contact email address:
We will only contact you for the specific purpose(s) you have agreed to in this survey. Take care not to enter any spaces after your email address.

____

Q9.9
If you agreed to be contacted in the preceding questions, please enter your name and contact phone number:

We will only contact you for the specific purpose(s) you have agreed to in this survey. This information will only be used if we contact you and will be kept separate from your survey responses.

End of Block: 9 Open-ended comments, demographic and contact details
