# Supplementary material for: Livestock Farm Recovery Following Bushfire in South-Eastern Australia: Impacts on Cattle and Sheep Health and Management
Source: Animals (Basel). 2025 Jun 14;15(12):1764. doi: 10.3390/ani15121764 (PMC12189280; doi:10.3390/ani15121764)
Supplement: Supplementary file 1 [file animals-15-01764-s001.zip › S3_Bushfire_farmadvisors.pdf]

Livestock Farm Recovery Following Bushfire in South-Eastern Australia: Impacts on Cattle and Sheep Health and Management

Supplement S3. Additional details about farm advisors during bushfire recovery on interviewed farms.

Professional advice to support recovery was sought by 45% (20/44) of interview respondents (two farms did not respond to this question). The types of professionals approached for advice are summarized in Table S3, 13 of the 20 farmers that sought advice utilised more than one type of advisor.

Table S3. Summary of advisors utilized in post-fire recovery period on 20 fire-affected farms with beef cattle and/or sheep in southeastern Australia, in the months following the bushfire in 2019–2020.

Advisor type	Number of Farms
Agronomist	14
Nutritional advice (farm advisors, nutritional consultants)	6
Veterinarians (animal health or nutrition advice)	5
Financial counsellors or banks	4
Government representative	2
Better Beef advisor	1
MLA Back to Business program	1
Company that owns the farm	1
